# Beta cell-specific PAK1 enrichment ameliorates diet-induced glucose intolerance in mice by promoting insulin biogenesis and minimising beta cell apoptosis

**DOI:** 10.1007/s00125-024-06286-2

**Published:** 2024-10-15

**Authors:** Miwon Ahn, Sangeeta Dhawan, Erika M. McCown, Pablo A. Garcia, Supriyo Bhattacharya, Roland Stein, Debbie C. Thurmond

**Affiliations:** 1 Department of Molecular and Cellular Endocrinology, Arthur Riggs Diabetes and Metabolism Research Institute, Beckman Research Institute, City of Hope, Duarte, CA USA; 2 Department of Translational Research and Cellular Therapeutics, Arthur Riggs Diabetes and Metabolism Research Institute, Beckman Research Institute, City of Hope, Duarte, CA USA; 3 Integrative Genomics Core, City of Hope, Duarte, CA USA; 4 Department of Molecular Physiology and Biophysics, Vanderbilt University, Nashville, TN USA

**Keywords:** Beta cell, Diet-induced obesity, Human islets, Insulin biogenesis, Insulin gene promoter, NEUROD1, PAK1, PDX1, Type 2 diabetes

## Abstract

**Aims/hypothesis:**

p21 (CDC42/RAC1) activated kinase 1 (PAK1) is depleted in type 2 diabetic human islets compared with non-diabetic human islets, and acute PAK1 restoration in the islets can restore insulin secretory function ex vivo. We hypothesised that beta cell-specific PAK1 enrichment in vivo can mitigate high-fat-diet (HFD)-induced glucose intolerance by increasing the functional beta cell mass.

**Methods:**

Human islets expressing exogenous PAK1 specifically in beta cells were used for bulk RNA-seq. Human EndoC-βH1 cells overexpressing myc-tagged PAK1 were used for chromatin immunoprecipitation (ChIP) and ChIP-sequencing (ChIP-seq). Novel doxycycline-inducible beta cell-specific PAK1-expressing (iβ*PAK1*-Tg) mice were fed a 45% HFD pre-induction for 3 weeks and for a further 3 weeks with or without doxycycline induction. These HFD-fed mice were evaluated for GTT, ITT, 6 h fasting plasma insulin and blood glucose, body composition, islet insulin content and apoptosis.

**Results:**

Beta cell-specific PAK1 enrichment in type 2 diabetes human islets resulted in decreased beta cell apoptosis and increased insulin content. RNA-seq showed an upregulation of *INS* gene transcription by PAK1. Using clonal human beta cells, we found that PAK1 protein was localised in the cytoplasm and the nucleus. ChIP studies revealed that nuclear PAK1 enhanced pancreatic and duodenal homeobox1 (PDX1) and neuronal differentiation 1 (NEUROD1) binding to the *INS* promoter in a glucose-responsive manner. Importantly, the iβ*PAK1*-Tg mice, when challenged with HFD and doxycycline induction displayed enhanced glucose tolerance, increased islet insulin content and reduced beta cell apoptosis when compared with iβ*PAK1*-Tg mice without doxycycline induction.

**Conclusions/interpretation:**

PAK1 plays an unforeseen and beneficial role in beta cells by promoting insulin biogenesis via enhancing the expression of *PDX1*, *NEUROD1* and *INS*, along with anti-apoptotic effects, that culminate in increased insulin content and beta cell mass in vivo and ameliorate diet-induced glucose intolerance.

**Data availability:**

The raw and processed RNA-seq data and ChIP-seq data, which has been made publicly available at Gene Expression Omnibus (GEO) at https://www.ncbi.nlm.nih.gov/geo/, can be accessed in GSE239382.

**Graphical Abstract:**

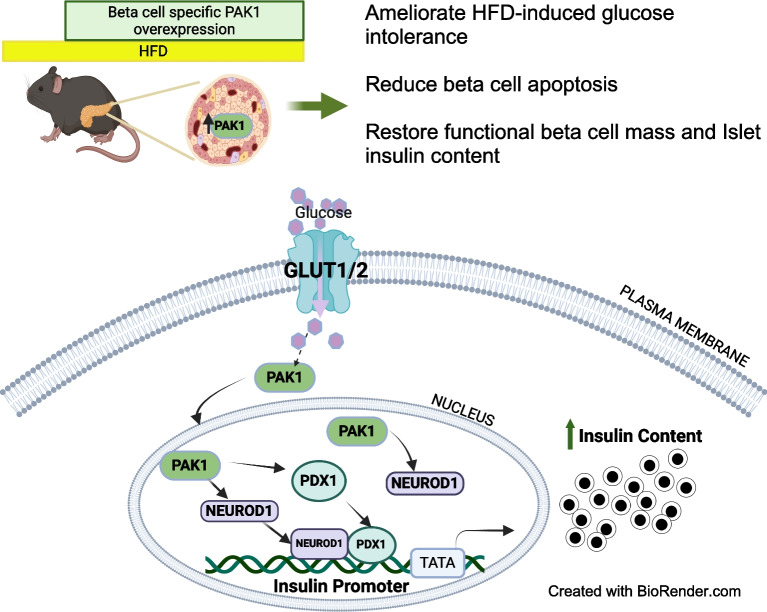

**Supplementary Information:**

The online version contains peer-reviewed but unedited supplementary material available at 10.1007/s00125-024-06286-2.


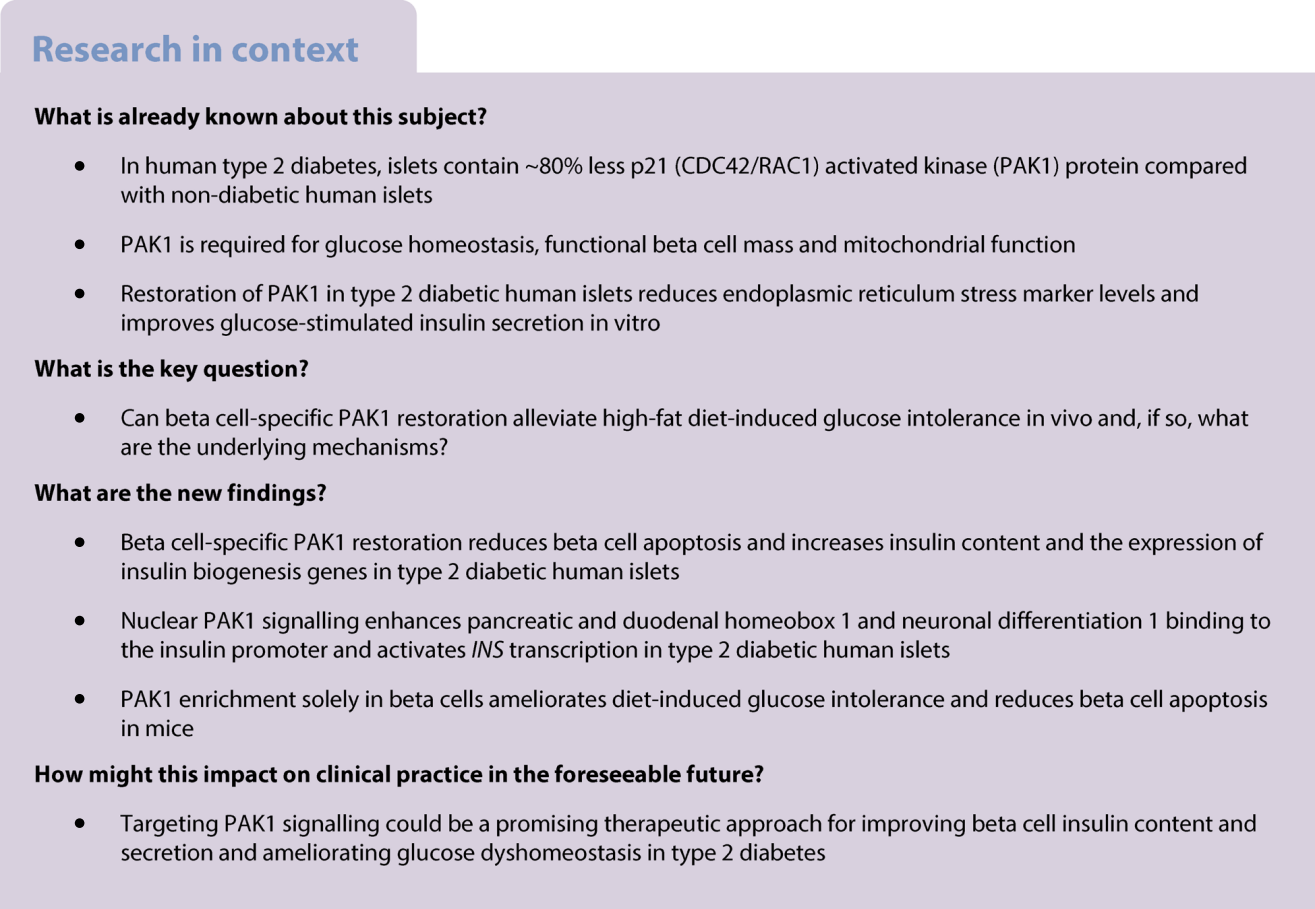


## Introduction

About 537 million adults are living with diabetes and 541 million adults are at risk of developing type 2 diabetes worldwide [1]. About 50% of individuals who have impaired glucose tolerance and/or fasting glucose progress to frank type 2 diabetes within 5 years [2]. Further, type 2 diabetes increases the risk of several other diseases such as atherosclerosis, nephropathy, neuropathy and retinopathy [1, 2].

Type 2 diabetes is a chronic metabolic disease characterised by impaired pancreatic islet insulin secretion and production, and peripheral insulin-stimulated glucose uptake (‘insulin action’). The combination of hyperglycaemia and glucolipotoxicity (GLT) promotes loss of beta cell mass via beta cell apoptosis and/or dedifferentiation, and leads to decreased insulin production and impaired insulin gene expression [3–6]. Insulin gene transcription involves more than 40 nuclear factors, with the canonical mediators being pancreatic and duodenal homeobox 1 (PDX1), neuronal differentiation 1 (NEUROD1) and MAF bZIP transcription factor A (MafA) [7]. Transcriptional regulation can involve a physical interaction between two factors that allosterically enhances their binding to DNA and/or the recruitment of cofactors to the gene [8–10]. The underlying mechanisms by which diabetogenic stimuli modulate the expression levels of these factors within the context of insulin gene expression and beta cell identity remain to be understood.

The human gene encoding p21 (CDC42/Rac1)-activated kinase 1 (PAK1) resides within the type 2 diabetes susceptibility locus on chromosome 11 [11]. PAK1 levels are reduced by ~80% in type 2 diabetic human islets, as well as in frank and impaired glucose tolerance mouse islets [12, 13]. We and others previously established the requirement for PAK1 in beta cell function and mass maintenance [14–17]. *Pak1*-knockout (KO) mice fed with a palmitate-rich high-fat diet (HFD) exhibit fasting hyperglycaemia and reduced beta cell mass [16]. Further, beta cell-specific inducible *Pak1*-KO (β*Pak1*-iKO) mice also exhibit glucose intolerance, beta cell dysfunction and increased beta cell apoptosis [14]. Overall, our prior work suggests an essential requirement for beta cell PAK1 in maintaining the functional islet beta cell mass.

PAK1 serves as an important signalling hub in response to metabolic stimuli that support insulin secretion and beta cell survival. PAK1 enhances beta cell survival [18] by inactivating the pro-apoptotic factor BCL2-associated agonist of cell death (BAD) [19] and activating the anti-apoptotic BCL2 apoptosis regulator (BCL2) [16]. Moreover, restoration of PAK1 in type 2 diabetic human islets reduces the hallmarks of endoplasmic reticulum stress, namely E74 like ETS transcription factor 2 (EIF2) phosphorylation and DNA-damaged inducible transcript 3 (CHOP) [14]. However, whether PAK1 enhancement in mice with impaired glucose tolerance carries the capacity to mitigate functional beta cell mass loss, and by what molecular mechanisms, remains to be elucidated.

Here, we used transcriptomics and chromatin immunoprecipitation sequencing (ChIP-seq) to investigate PAK1 mechanisms using PAK1-enriched human type 2 diabetic islets and beta cells. Further, we addressed whether beta cell-specific PAK1 induction in vivo could reverse HFD-induced glucose intolerance and hyperglycaemia, and deciphered the mechanisms involved.

## Methods

Please refer to the electronic supplementary materials (ESM) Methods for details.

### Human islets

Pancreatic type 2 diabetic and non-diabetic (ND) human islets were obtained through the Integrated Islet Distribution Program and the City of Hope Islet Core (donor information is in electronic supplementary material [ESM] Table 1). Adenoviral PAK1 overexpression using a rat insulin promoter (RIP) in human islet beta cells was as described previously [16]. For bulk RNA-seq, PAK1-transduced ND and type 2 diabetic human islets were used. Perifusion analysis of PAK1-transduced ND human islets was as described previously [20]. See ESM Methods for details. The checklist for reporting human islet preparations is provided in the ESM.

### Animals

Previously established conditional beta cell-specific *Pak1*-knockout (β*Pak1*-iKO) mice were used [14]. Mice with floxed *Pak1* alleles (*Pak1*^fl/fl^ mice) [21] were crossed with tamoxifen-inducible transgenic mice containing the mouse insulin promoter (MIP)-*Cre*^+/ERTM^ (obtained from L. Philipson, University of Chicago, IL, USA). To ablate beta cell-specific PAK1, 9- to 10-week-old male mice were administered tamoxifen via oral gavage for five consecutive days (2 mg/40 g of body weight). All mice were randomised based on sex and genotype from multiple litters and cages. Vehicle (corn oil) was provided to control (Ctrl) mice to prevent induction. Islets from 16- to 18-week-old mice were used for quantification of insulin content and insulin granules using transmission electron microscopy (see ESM Methods for details). All samples were tested in a blinded manner. To generate beta cell-specific PAK1-overexpressing transgenic (iβ*PAK1*-Tg) mice with PAK1-enrichment in beta cells, Tet response element-h*Pak1* (TRE-h*PAK1*) mice were initially made by C57BL6/J (Strain no. #000664, The Jackson Laboratory, USA; https://www.jax.org/strain/000664) blastocyst injection at the Indiana University School of Medicine Transgenic Core (IN, USA); only one founder transmitted the transgenes. Two additional founder lines were generated at the City of Hope Transgenic Core (CA, USA). TRE-h*PAK1* mice were crossed with beta cell-specific RIP-rtTA mice (strain no. #008250, The Jackson Laboratory, USA; https://www.jax.org/strain/008250) and the progeny were used in the HFD study. All animal experiments were approved by the Institutional Animal Care and Use Committees at Indiana University School of Medicine (IN, USA) and City of Hope (CA, USA).

### Cell lines

EndoC-βH1 cells obtained from Human Cell Design (Toulouse, France), cultured as described previously [22], were transiently transfected with cytomegalovirus promoter (pCMV)-myc-h*P*AK1 plasmid using lipofectamine 2000 (Thermo Fisher Scientific, IL, USA), harvested and used for chromatin immunoprecipitation sequencing (ChIP-seq) and immunoblot analyses. For ChIP-seq, EndoC-βH1 cells were transduced (100 multiplicity of infection [MOI] of AdCMV-myc-h*PAK1*) for 2 h, washed with PBS and incubated for 48 h. INS1 832/13 (gift from C. B. Newgard, Duke University, Durham, NC) cell culture and EndoC-βH1/INS1 832/13 siRNA PAK1 studies are detailed in ESM Methods. All cell lines were tested for mycoplasma every 3 months using Mycoplasma PCR ELISA (Millipore, MA, USA) and all tested negative. The cell lines were authenticated using immunostaining and assessed for insulin content (see below).

### Insulin content

Insulin ELISA or ultrasensitive human insulin RIA kit (Millipore, MA, USA) was used to quantify insulin protein content in human and mouse islets. See ESM Methods for details.

### Quantitative PCR

See ESM Methods for details of quantitative PCR (qPCR) using QuantiTech one-step SYBR Green PCR Kit (Qiagen, CA, USA). Gene expression was quantified using the ${2}^{{-\Delta \Delta \text{C}}_{\text{t}}}$ method and normalised to tubulin (housekeeping gene). The primers are listed in ESM Table 2.

### Immunoblotting

See ESM Methods for details of antibodies against PAK1(RRID: AB_330222), Myc (RRID: AB_11159758), TATA-box binding protein (TBP, RRID: AB_2799258), PDX1(RRID: AB_10706174), NEUROD1 (RRID: AB_10549071), BCL2 (RRID: AB_309943), tubulin (RRID: AB_2617116) and cleaved caspase 3 (CC-3, RRID: AB_2341188).

### RNA-seq and ChIP-seq analysis

RNA-seq raw fastq reads were aligned to the human hg19 genome using TopHat2 version 2.1.2 [23]. Gene counts were obtained through htseq-count [24], utilising UCSC known gene annotations (TxDb.Hsapiens.USCS.hg19.knownGene) [25], and converted into counts per million (CPM) using edgeR [26, 27]. Human islet genes with CPM of ≥1 in ≥1 sample were retained. To determine differentially expressed genes (DEGs) between PAK1-enrichment and Ctrl, limma-voom version 3.58.1 was used while factoring donor-specific effects into the design equation [28, 29]. Positive or negative normalised enrichment score (NES) served as an indicator that most genes in this pathway are upregulated or downregulated, respectively. For ChIP-seq, chromatin from myc-h*PAK1*-transfected EndoC-βH1 cells was immunoprecipitated with myc-PAK1 and the resulting DNA was used to construct libraries, sequenced with the Illumina genome Analyzer (Illumina, CA, USA). The PAK1 peaks were identified in each replicate using MACS version 2.1.0 [30] and annotated based on genomic region using the R package Annotator version 1.28.0 [31]. To compare the peak read densities between the two replicates, the tag count of the larger sample was reduced by random sampling to match the number of tags present in the smaller sample. Peaks with mean read density between the two replicate >1 SD above the mean were used. See ESM Methods for details.

### ChIP assay

PAK1-transduced EndoC-βH1 cells were carried out using the micro-ChIP protocol [32, 33]. Antibodies against PDX1 (goat, a kind gift from C. V. E. Wright, Vanderbilt University, TN, USA), NEUROD1 and IgG were used. qPCR was performed to amplify the DNA fragment containing the *INS* promoter region covering the PDX1- and NEUROD1-binding sites, with primers listed in ESM Table 2. See ESM Methods for details.

### HFD study

Eight-week-old iβ*PAK1*-Tg male mice, on a pure C57BL6/J genetic background, were fed an HFD (no. D01030108, Research Diet, USA) for 3 weeks; female mice were not included given that B6 females lack response to short-term acute 45% HFD. Following a GTT, the most glucose-intolerant mice (experimental group) continued to be fed the HFD with doxycycline (Dox; 625 mg/kg) for a further 3 weeks. The remaining Ctrl (TRE-h*PAK1*; RIPrtTA) double-transgenic (dTg) mice continued on the HFD without Dox until the end of the experiments. A third group consisting of single-transgenic (sTg) TRE-h*PAK1* 8-week-old male mice were maintained on the standard chow diet (LabDiet 5053; Rancho Cucamonga, CA, USA) and treated with Dox at the same time as the experimental group for 3 weeks. A GTT was conducted at the conclusion of the 3 weeks of Dox treatment. All HFD-fed mice were housed individually on Sani-ChIP bedding. The food was changed and measured twice weekly and body weight was measured once a week. Harvested pancreatic islets [34] were evaluated for functional beta cell mass via static-culture glucose-stimulated insulin secretion (GSIS) assays, insulin content and western blot. See ESM Methods for details.

### GTT, ITT, fasting blood glucose and plasma insulin content

GTT, ITT and measurement of fasting blood glucose and plasma insulin content were performed on HFD- or chow-fed mice fasted for 6 h (08:00–14:00 hours) as described previously [14]. See ESM Methods for details.

### Whole-body composition analysis

Whole-body composition (fat and lean mass) was determined using quantitative magnetic resonance technology (EchoMRI 3-in-1; Echo Medical Systems, Houston, TX, USA).

### TUNEL staining and morphometric assessment of islets

The TUNEL in situ TMR-Red Cell Death Detection Kit (Sigma-Aldrich, St Louis, MO, USA) was used as previously described [14]. The percentage of TUNEL^+^ cells relative to the total number of insulin^+^ beta cells was quantified. Mouse islet morphometry was evaluated using anti-insulin immunofluorescence staining of pancreatic sections as before [35]. All samples were tested in a blinded manner. See ESM Methods for details.

### Statistical analyses

Data are presented as means ± SEM. Statistical analysis was performed using GraphPad Prism 9 (GraphPad Software, La Jolla, CA, USA). All data were evaluated for statistical significance by two-tailed Student’s *t* test for comparison of two groups or by ANOVA Tukey or Fisher’s least significant difference (LSD) test for more than two groups. Statistical information, including the value of *n*, statistical test used and significance (*p* value), is reported in the figures and their legends. Data points that fell outside the acceptable range were excluded from analysis.

## Results

### Beta cell-specific PAK1 depletion leads to reduced insulin granule number and content

**Fig. 1:**
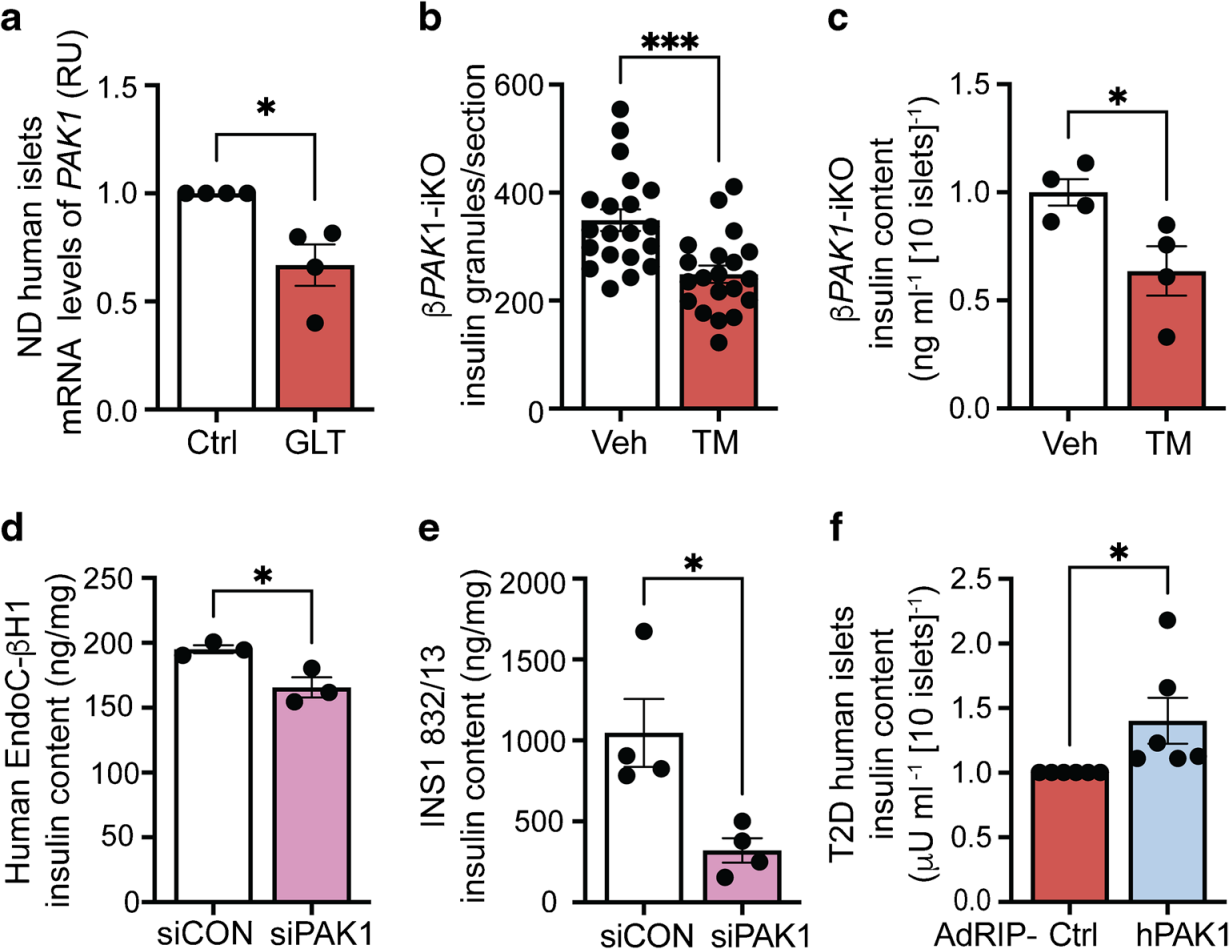
PAK1 depletion is correlated with reduced insulin granule numbers and insulin content. (**a**) Quantification of *PAK1* mRNA in ND human islets (*n*=4 donors) exposed to GLT stress for 48 h. (**b**, **c**) Quantification of insulin granule numbers (**b**) and insulin content (**c**) in tamoxifen-treated β*PAK1*-iKO mouse islets vs vehicle-treated Ctrl mice. Insulin granules were counted in a blinded manner using transmission electron microscopy of isolated islets from β*PAK1*-iKO and Ctrl mice. Total 20 sections, *n*=3 (**b**) or *n*=4 (**c**) mice/treatment group. (**d**, **e**) Quantification of insulin content in PAK1-depleted clonal human EndoC-βH1 (**d**) and rat INS1 832/13 cell lines (**e**); *n*=3 (**d**) or *n*=4 (**e**) independent experiments. (**f**) Quantification of insulin content in type 2 diabetic human islets following adenovirus-mediated restoration of beta cell-specific PAK1 (*n*=6 donors/vector). All data are shown as the mean ± SEM and presented in a bar graph. *p<0.05, ***p<0.001 (unpaired two-tailed Student’s *t* test). AdRIP-Ctrl, Ctrl empty vector; AdRIP-hPAK1, human PAK1 vector; RU, relative units; siCON, Ctrl siRNA; siPAK1, *PAK1* siRNA; T2D, type 2 diabetes; TM, tamoxifen; Veh, vehicle

Our prior work established that PAK1 levels are reduced in type 2 diabetic human islets and in ND human islets exposed to diabetogenic stressors (e.g. GLT) [13, 16]. To determine whether diabetogenic stressors also affect *PAK1* mRNA levels, ND human islets were subjected to GLT (16.7 mmol/l glucose and 0.5 mmol/l palmitate) for 48 h. A 40% reduction in *PAK1* mRNA levels was observed in GLT-treated vs vehicle-treated islets (Fig. 1a).

We previously reported that islets from conditional tamoxifen-inducible β*Pak1*-iKO mice have blunted GSIS [14]. To determine PAK1 requirement in regulating beta cell responsiveness, we quantified the insulin granule number and insulin protein content in β*Pak1*-iKO mouse islets. The number of insulin granules and total insulin content in β*Pak1*-iKO mouse islets was significantly reduced vs *flox/flox*; *Cre*^+/−^ vehicle-induced Ctrl islets (Fig. 1b, c). Similarly, insulin content was reduced in human and rat clonal beta cells post-PAK1 knockdown (Fig. 1d, e) but with differing efficiency (33.8 ± 1.4% and 60.5 ± 5.7% reduction for EndoC-βH1 and INS1 832/13 cells, respectively). Conversely, adenoviral beta cell-specific PAK1 enrichment significantly increased insulin content in type 2 diabetic human islets (Fig. 1f). These data suggest that PAK1 is required for insulin biogenesis and that PAK1 enrichment in type 2 diabetic human islet beta cells is sufficient to increase insulin content.

### Beta cell-specific PAK1 enrichment in type 2 diabetic human islets increases key insulin biogenesis activators

**Fig. 2:**
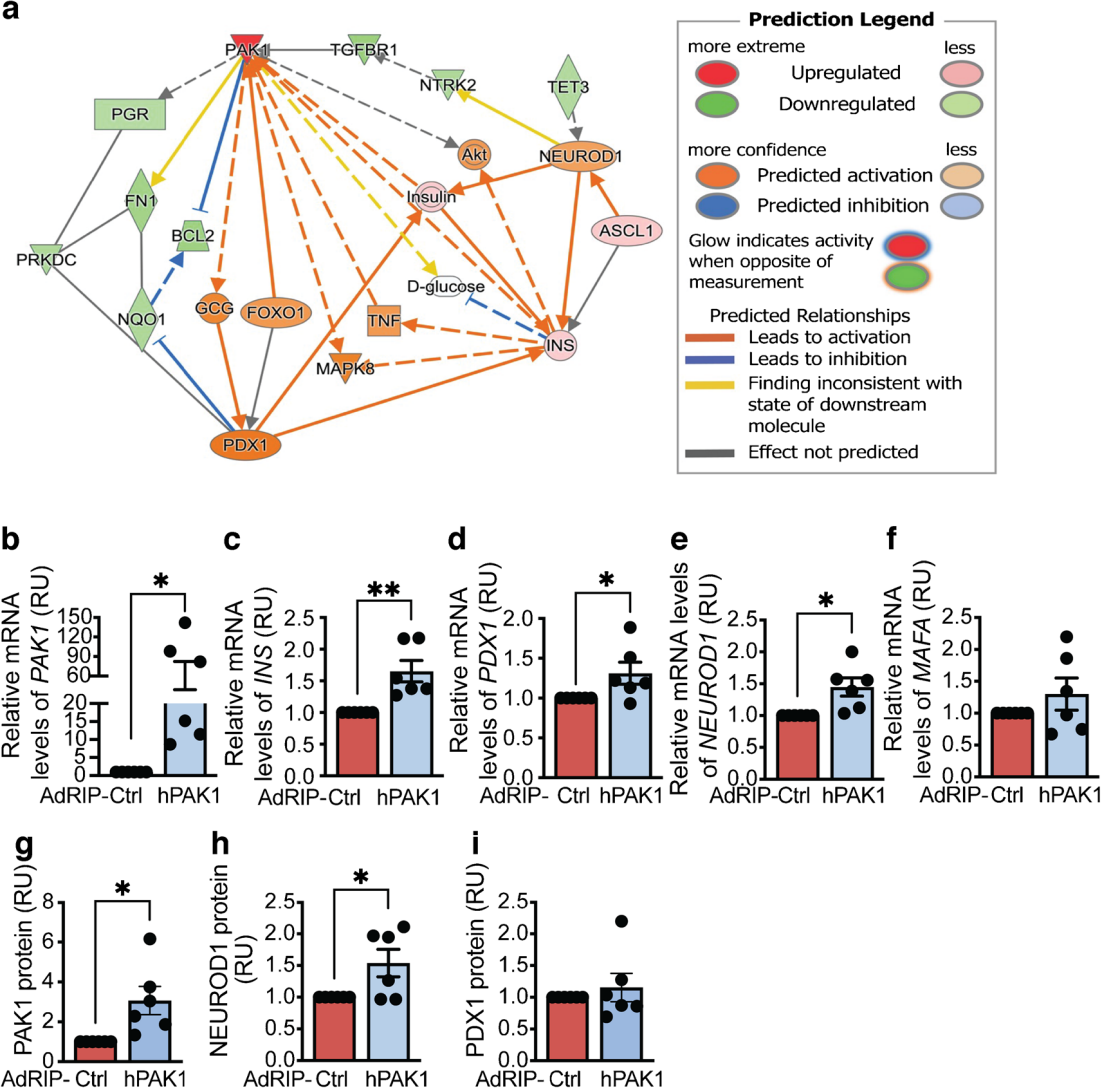
Beta cell-specific PAK1 enrichment impacts *INS*, *PDX1* and *NEUROD1* mRNA levels, and NEUROD1 protein levels in type 2 diabetic human islets. Type 2 diabetic human islets were transduced with human PAK1 (AdRIP-hPAK1) or empty control vector (AdRIP-Ctrl). (**a**) Bulk RNA-seq data from transduced type 2 diabetic islets were used to generate an IPA network (*n*=3 donors/vector). (**b**–**f**) qPCR analysis of *PAK1* (**b**), *INS* (**c**), *PDX1* (**d**), *NEUROD1* (**e**) and *MAFA* (**f**) mRNA levels in transduced type 2 diabetic islets (*n*=6 donors/vector). (**g**–**i**) Protein levels of PAK1 (**g**), NEUROD1 (**h**) and PDX1 (**i**) were determined by immunoblot (*n*=6 donors/vector). All data are shown as mean ± SEM and presented in a bar graph. *p<0.05, **p<0.01 (unpaired two-tailed Student’s *t* test). RU, relative units

To determine pathways and genes downstream of *PAK1* that potentially modulate insulin biogenesis, we performed bulk RNA-seq using type 2 diabetic human islets adenovirally transduced to overexpress PAK1 (adenovirus rat insulin promoter [AdRIP]-h*PAK1*), which increases PAK1 protein by about fourfold [14], or the empty vector Ctrl. We identified 312 DEGs with *p*<0.05 in PAK1-overexpressing vs Ctrl transduced human type 2 diabetic islets (ESM Table 3). The top ten altered genes are presented in ESM Fig. 1a. Gene ontology (GO) analysis was performed to define the biological processes enriched by PAK1 overexpression in human type 2 diabetes islets (ESM Fig. 1b). Dysfunctional type 2 diabetic human islets exhibit a depletion of critical beta cell factors (MafA, MafB and PDX1) [36]. The MafB transcription factor is a distinct characteristic of primate islet beta cells and a critical regulator of beta cell identity in humans [37]. While there was a significant increase in *INS* levels in the PAK1-enriched type 2 diabetic human islets when compared with the Ctrls, RNA-seq showed no change in *MAFB* levels (onefold change, PAK1-enriched vs Ctrl) (ESM Table 4). Ingenuity pathway analysis (IPA) pointed to elevated mRNA levels of the key insulin biogenesis genes, *PDX1* and *NEUROD1* (Fig. 2a). qPCR validated the elevated *PAK1*, *INS*, *PDX1* and *NEUROD1* mRNA levels in the AdRIP-hPAK1 human type 2 diabetic islets (Fig. 2b–e). However, *MAFA* was not significantly changed in PAK1-transduced islets (Fig. 2f). PAK1 overexpression affected PAK1 and NEUROD1 (Fig. 2g, h) but not PDX1 protein levels (Fig. 2i).

### PAK1 enrichment enhances PDX1 and NEUROD1 occupancy at the human *INS* promoter in response to high glucose

**Fig. 3:**
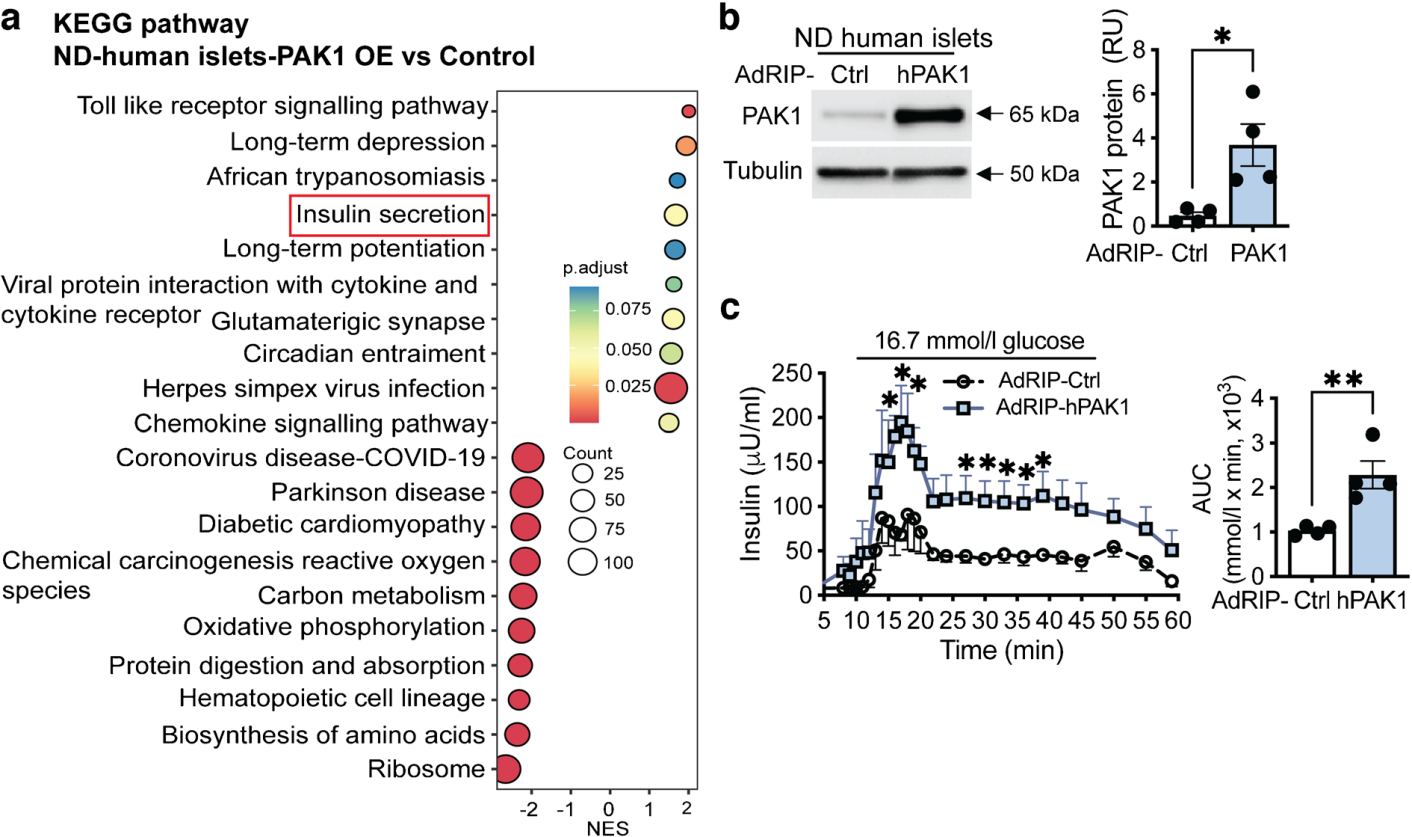
Beta cell-specific overexpression of PAK1 enhances insulin secretion in ND human islets. ND human islets were adenovirally transduced with AdRIP-Ctrl or AdRIP-*hPAK1* to yield beta cell-specific PAK1 overexpression. (**a**) Bulk RNA-seq data from transduced ND human islets were analysed by the KEGG pathway (*n*=4 donors). (**b**) PAK1 protein levels were determined by immunoblot and quantified. All data are shown as mean ± SEM. The quantification data are presented as a bar graph. *p<0.05 (unpaired two-tailed Student’s *t* test). (**c**) Human islets were perifused with 2.8 mmol/l (0–10 min and 46–60 min) and 16.7 mmol/l (11–45 min) glucose; insulin content and AUC are shown. Curves represent the mean average ± SEM of four independent sets of human donor islets performed in paired experiments and the AUC data are presented as a bar graph, *p<0.05 (two-way ANOVA, Tukey test), **p<0.01 (unpaired two-tailed Student’s *t* test)

Our RNA-seq analysis revealed that PAK1 overexpression increased mRNA levels of genes related to insulin biogenesis in type 2 diabetic human islets. Given the heterogeneous and progressive nature of type 2 diabetes, we evaluated PAK1 regulation of insulin biogenesis-related genes under baseline conditions and performed RNA-seq analysis using PAK1-overexpressing ND human islets. We identified 420 DEGs (*p*<0.05) (ESM Table 4); the heatmap displayed genes affected by PAK1 overexpression (ESM Fig. 1c). RNA-seq analysis revealed that genes related to insulin biogenesis were unaffected. We subjected these DEGs to gene set enrichment analysis (GSEA) using GO-biological process (GO-BP). The top ten enriched pathways from GSEA (ESM Fig. 1d) were ranked by NES, which measures the degree of enrichment of the pathway among the DEGs. GO analyses revealed upregulation of chromatin remodelling and organisation pathways in PAK1-enriched ND islets (ESM Fig. 1d). Furthermore, Kyoto Encyclopedia of Gene and Genomes (KEGG) pathway analysis showed an upregulation of insulin secretion genes (Fig. 3a). Consistent with this, performing islet perifusion on PAK1-transduced ND islets showed an increase in GSIS (Fig. 3b, c). Therefore, PAK1 overexpression can upregulate chromatin remodelling and organisation, leading to improved functional beta cell mass in ND human islets.

**Fig. 4:**
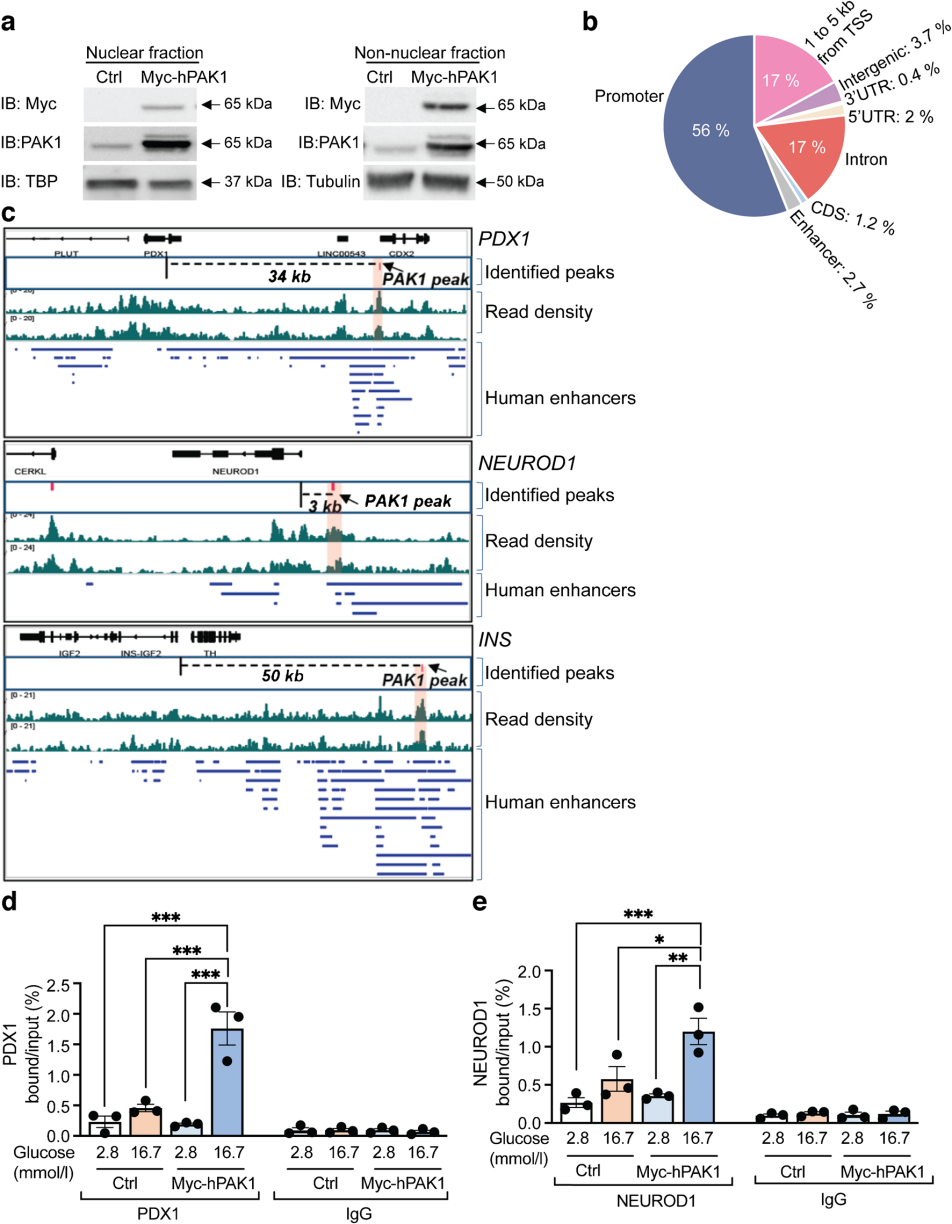
PAK1 enrichment in human clonal beta cells impacts PDX1 and NEUROD1 occupancy at the *INS* promoter under high-glucose conditions. (**a**) Representative immunoblots of nuclear and non-nuclear fractions (*n*=3 independent experiments) from myc-hPAK1 transfected human EndoC-βH1 cells. (**b**) Distribution of myc-hPAK1 binding sites in human EndoC-βH1 cells relative to genomic landmarks. (**c**) PAK1 bound *INS*, *PDX1* and *NEUROD1* in the enhancer region. (**d**, **e**) Quantification of PDX1 (**d**) and NEUROD1 (**e**) occupancy at the *INS* promoter in indicated EndoC-βH1 cells under basal (2.8 mmol/l) or high (16.7 mmol/l) glucose conditions (*n*=3 independent experiments). Data show means ± SEM and presented in a bar graph. *p<0.05, **p<0.01, ***p<0.001 (one-way ANOVA, Tukey test). CDS, coding sequence; TSS, transcription start site; UTR, untranslated region

PAK1 can localise to the nuclear and non-nuclear compartments in other cell types [38]. Hence, we hypothesised that PAK1 could localise to the beta cell nucleus. We examined nuclear and non-nuclear fractions from EndoC-βH1 cells transiently expressing myc-tagged human PAK1 (myc-hPAK1). Myc-hPAK1 was detected in the nuclear fractions by immunoblot (Fig. 4a). We validated the purity of fractions using TBP (nuclear marker) and tubulin by immunoblot (ESM Fig. 2). Thus, we hypothesised that nuclear PAK1 functions in insulin biogenesis. We conducted a genome-wide PAK1 chromatin occupancy analysis through ChIP-seq using myc-hPAK1-expressing EndoC-βH1 cells. Most of the myc-hPAK1 (56%) occupied promoter regions; other notable myc-hPAK1-bound genomic regions included transcription start sites (17%), introns (17%), intergenic regions (3.7%) and enhancers (2.7%) (Fig. 4b). Interestingly, ChIP-seq revealed PAK1 occupancy at the *INS*, *PDX1* and *NEUROD1* enhancer regions (Fig. 4c).

We performed IPA to discern the functional categories represented by PAK1 target genes. Consistent with the known functions of PAK1, this analysis showed significant association with molecular and cellular functions in cell death and survival, and RNA post-transcriptional modification (ESM Fig. 3a). Further, we performed hypergeometric optimisation of motif enrichment (HOMER) analysis using PAK1-bound genomic regions [39]; *PRDM10*, *ZBTB33*, *HINFP*, *NFATC1* and *NKX3-2* were identified as prominent motifs enriched in PAK1-occupied regions (ESM Fig. 3b).

Given that PAK1 overexpression increased *PDX1* and *NEUROD1* mRNA levels and insulin content, we tested whether PAK1 would affect the association of PDX1 and NEUROD1 with the *INS* promoter. Glucose upregulates insulin gene transcription within 30 min [40, 41]. For this, we performed a ChIP assay using myc-hPAK1-expressing and Ctrl EndoC-βH1 cells stimulated with 16.7 mmol/l glucose for 30 min. We found that glucose-stimulated cells overexpressing PAK1 displayed increased PDX1 and NEUROD1 enrichment at the *INS* promoter (Fig. 4d, e). These results suggest that nuclear PAK1 increases the binding of corresponding proteins to the *INS* promoter in a glucose-responsive manner. To determine whether PAK1 overexpression alters insulin biogenesis gene expression under basal conditions (5.5 mmol/l glucose), real-time PCR was performed using PAK1-transduced EndoC-βH1 cells. The expression of insulin biogenesis genes (*INS*, *PDX1*, *NEUROD1*, *MAFA1* and *MAFB)* under basal conditions was unaltered with PAK1 overexpression (ESM Fig. 4). Thus, PAK1-dependent increase in insulin biogenesis genes may be context-dependent, as observed in type 2 diabetes. Furthermore, the glucose-inducible recruitment of PAK1 to genomic regions relevant to the transcriptional control of insulin biogenesis genes supports this notion.

### Beta cell-specific PAK1-overexpression in mice abrogates HFD-induced glucose intolerance in vivo

**Fig. 5:**
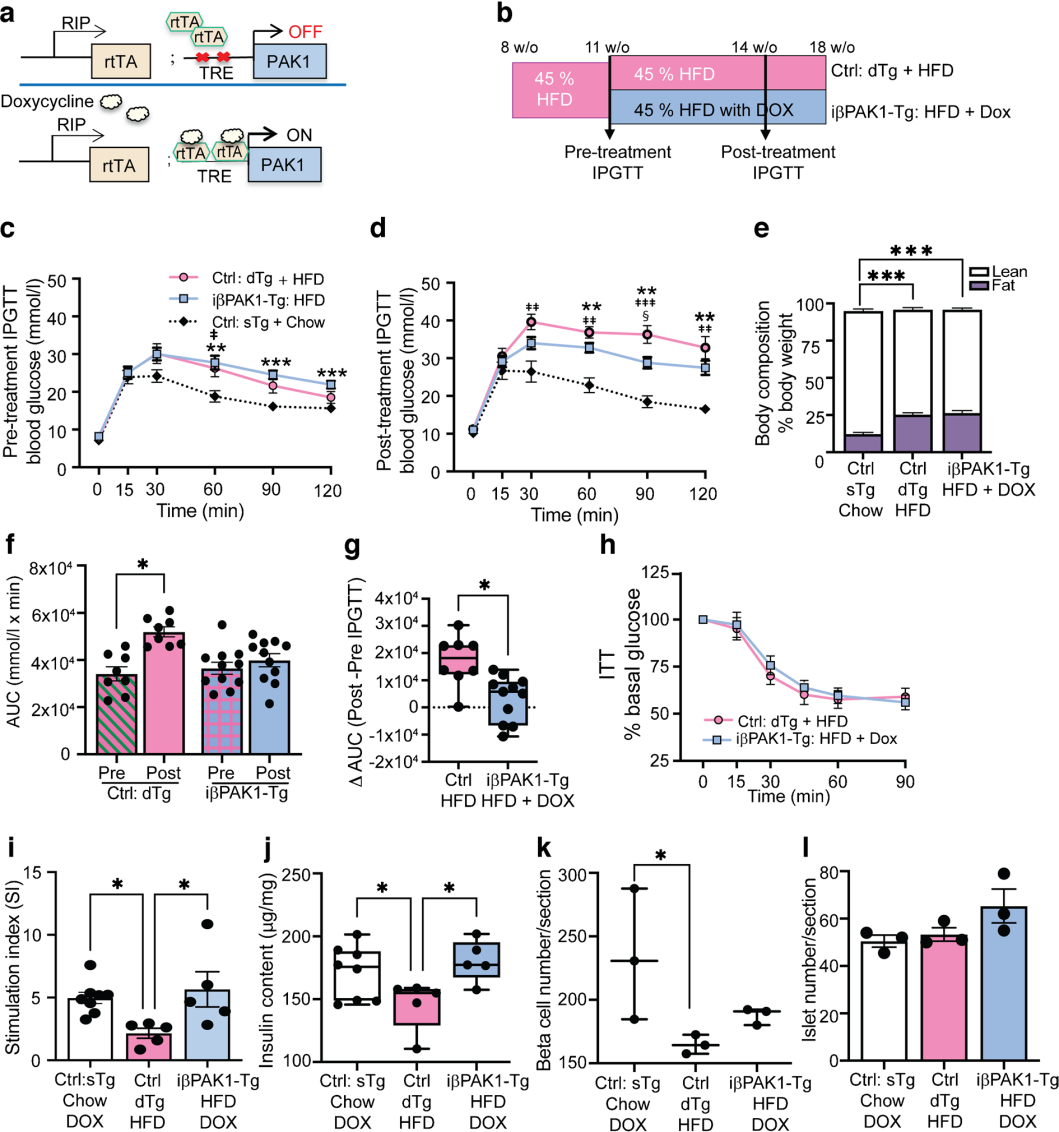
Beta cell-specific PAK1 enrichment ameliorates glucose intolerance in HFD-fed PAK1-expressing transgenic mice. (**a**) Schematic of beta cell-specific Dox-inducible PAK1-expressing mice used. (**b**) Schematic of the HFD study design. (**c**, **d**) GTT analyses at pre-treatment (**c**) and post-treatment (+Dox) (**d**). (**e**) Body composition analysis post-treatment. (**f**) AUC analyses from GTT data from (**c**, **d**). (**g**) Differential AUC from post-treatment GTT AUC-pre-treatment GTT AUC per mouse. (**h**) ITT over 90 min. (**c**–**h**) Ctrl: dTg + HFD, *n*=8; iβPAK1-Tg: HFD + Dox, *n*=11; Ctrl: sTg + Chow + Dox, *n*=7. (**i**) Static-culture insulin secretion assays in which islets were incubated in 2.8 mmol/l glucose or 16.7 mmol/l glucose. Data are shown as stimulation index: 16.7 mmol/l glucose-stimulated/2.8 mmol/l glucose-stimulated insulin. (**j**) Quantification of islet insulin content. (**i**, **j**) Ctrl: dTg + HFD, *n*=5; iβPAK1-Tg: HFD + Dox, *n*=5; Ctrl: Chow + Dox, *n*=7. (**k**, **l**) Total insulin^+^ beta cells (**k**) and total islet number (**l**) (quantified across >9 pancreas sections per group, *n*=3/group). Data are expressed as means ± SEM except for box and whisker plots (**g**, **j**, **k**), which show median and minimum to maximum, and all show data points. Statistics: two-way ANOVA Tukey test (**c**, **d**, **f**); one-way ANOVA Tukey test (**e**, **i**, **l**); one-way ANOVA Fisher’s LSD test (**j**, **k**); unpaired two-tailed Student’s *t* test (**g**). In (**c**, **d**) ^‡^Ctrl: dTg + HFD vs Ctrl: sTg + Chow (without Dox in **c**, with Dox in **d**); *iβPAK1-Tg: HFD vs Ctrl: sTg + Chow (without Dox in **c**, with Dox in **d**); ^§^Ctrl: dTg + HFD vs iβPAK1-Tg: HFD + Dox. In (**e**–**g**, **i**–**k**) *as indicated. Single symbols ^‡^,*,^§^*p*<0.05; double symbols ^‡‡^,**p<0.01; triple symbols ^‡‡‡^,***p<0.001. w/o, weeks old

To evaluate the ability of beta cell PAK1 enrichment to mitigate the negative effects of HFD-induced glucose intolerance and loss of functional beta cell mass in vivo, we used a novel Dox-inducible mouse model to express PAK1 in a beta cell-selective manner (Fig. 5a). Dox treatment induced exogenous PAK1 expression in the iβ*PAK1*-Tg mouse islets vs untreated islets (ESM Fig. 5a), and hypothalamic PAK1 levels were unaltered by Dox induction, confirming beta cell specificity of PAK1 enrichment (ESM Fig. 5a). The iβ*PAK1*-Tg mice displayed enhanced glucose tolerance in a GTT assay vs Ctrl (ESM Fig. 5b), without changes to peripheral insulin tolerance (ESM Fig. 5c). This phenotype was reproducibly detected in all three mouse founder lines (ESM Fig. 5d–g). After validating the mouse model, we developed a glucose intolerance rescue paradigm to determine whether HFD-induced glucose intolerance could be reversed/mitigated/ameliorated in iβ*PAK1*-Tg mice (Fig. 5b).

In this rescue paradigm, 8-week-old dTg male mice were fed an HFD only for 3 weeks and then evaluated for glucose intolerance (pre-treatment GTT; Fig. 5c). Mice with the most severe glucose intolerance were fed HFD + Dox to induce islet beta cell PAK1 expression. The Ctrl dTg mice were maintained on an HFD only. After 3 weeks, all mice were subjected to post-treatment GTT. The HFD + Dox iβ*PAK1*-Tg mice exhibited reduced glucose intolerance vs Ctrl HFD-fed mice (Fig. 5d). The HFD was effective, as shown by the significantly elevated proportion of body fat observed in 18-week-old HFD-Ctrl dTg and HFD-iβ*PAK1*-Tg mice vs chow-fed mice (Fig. 5e). Both HFD groups showed similar increases in body weight and energy intake (ESM Fig. 6a, b). AUC analysis (0–120 min) pre- and post-treatment showed that Ctrl mice exhibited elevated glucose intolerance post-GTT, whereas HFD + Dox iβ*PAK1*-Tg mice resisted this elevation, despite continuing to consume the HFD (Fig. 5f, g). No differences in the ITT were seen when comparing HFD-Ctrl dTg mice with iβ*PAK1*-Tg mice at 16 weeks of age (Fig. 5h).

At 15 weeks of age, HFD + Dox iβ*PAK1*-Tg mice and healthy chow-fed mice had similar fasting (6 h) plasma insulin levels (ESM Table 5); fasting hyperinsulinaemia was detected in the Ctrl HFD-fed mice, as shown previously [42]. All three groups had similar fasting glucose levels (ESM Table 5), suggesting that the elevated plasma insulin in the HFD-dTg group effectively compensated and managed blood glucose levels in this short-term HFD paradigm. Tissue weights were similar among the HFD groups at 18 weeks of age (ESM Table 6). The HFD groups displayed significantly elevated fasting cholesterol levels vs the chow-fed group; no differences were observed between the two HFD groups (ESM Table 7). Further, no intergroup differences in fasted serum triglycerides or NEFA were observed (ESM Table 7).

Consistent with PAK1-enriched type 2 diabetic human islets, HFD-fed iβ*PAK1*-Tg mouse islets demonstrated restoration of GSIS vs chow + Dox-fed Ctrl mice fed (Fig. 5i). Additionally, the islets of iβ*PAK1*-Tg mice displayed significantly higher insulin content vs HFD-Ctrl islets (Fig. 5j). HFD-Ctrl mice exhibited significantly reduced insulin content and beta cell number vs chow + Dox-fed Ctrl mice (Fig. 5j, k), while there was no significant difference in the number of islets (Fig. 5l). These data indicate that PAK1 enrichment in beta cells alone is sufficient to deter HFD-induced glucose intolerance in vivo, corresponding with restored functional beta cell mass and elevated islet insulin content.

### HFD-challenged iβ*PAK1*-Tg mouse islets have reduced beta cell apoptosis

**Fig. 6:**
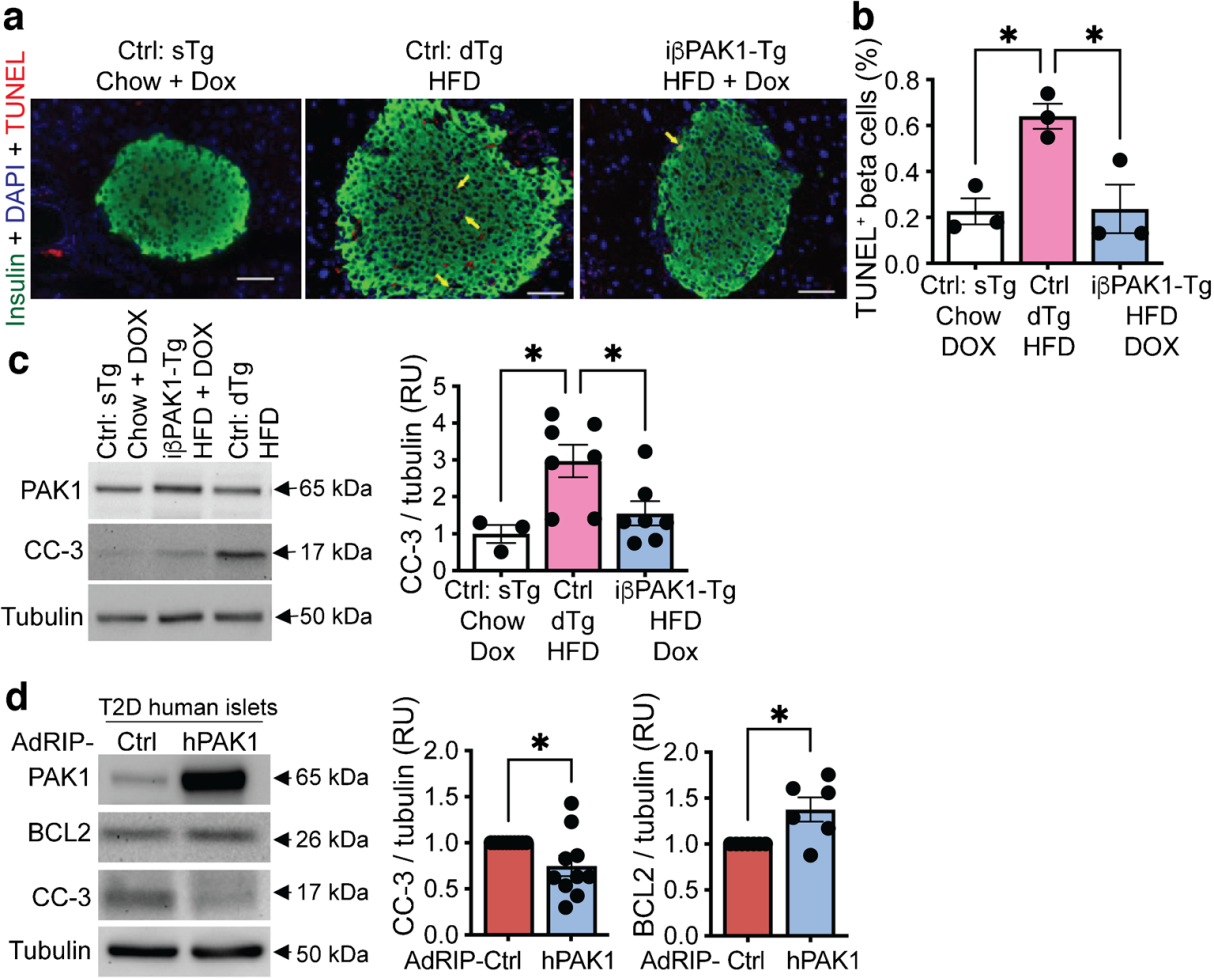
Beta cell-specific PAK1 restoration impacts beta cell apoptosis in transgenic mouse and type 2 diabetic human islets. (**a**) Representative TUNEL immunofluorescence image from indicated mouse islets. Yellow arrow indicates double positive beta cells for TUNEL (red) and insulin (green) (*n*=3 mice). Scale bar, 50 μm. (**b**) Quantification of TUNEL^+^ beta cells in indicated mouse islets (*n*=3 mice). (**c**) Representative immunoblot of CC-3 and PAK1 post-Dox treatment in indicated mouse islets (Ctrl: sTg + Chow + Dox, *n*=3; Ctrl: dTg+ HFD, *n*=7; iβ*PAK1*-Tg: HFD + Dox, *n*=7). The bar graph displays the quantification of CC-3 immunoblot relative to tubulin in relative units (RU). (**d**) Representative immunoblot of PAK1-enriched type 2 diabetic human islets for BCL2 (*n*=6 donors), CC-3 and PAK1 (*n*=10 donors). Data in (**b**–**d**) are shown as mean ± SEM and presented as a bar graph. *p<0.05 (unpaired two-tailed Student’s *t* test)

To determine whether the observed elevations of insulin content and islet beta cell number of the HFD-fed iβ*PAK1*-Tg mice correlated with reduced beta cell death, mice were maintained on HFD for an additional 4 weeks to facilitate the detection of apoptotic cells. Pancreases from HFD-fed 18-week-old iβ*PAK1*-Tg mice showed significantly reduced TUNEL^+^ beta cells vs HFD-fed Ctrl mouse pancreases (Fig. 6a, b). Consistent with this, HFD-fed iβ*PAK1*-Tg mouse islets displayed lower levels of CC-3 (apoptotic marker) and showed PAK1 protein enrichment vs HFD-fed Ctrl mouse islets (Fig. 6c). Further, exogenous PAK1 induction reduced CC-3 and increased BCL2 (anti-apoptotic marker) in type 2 diabetes human islets (Fig. 6d). These findings support the concept that beta cell-PAK1 enrichment leads to increased functional beta cell mass via an anti-apoptotic effect.

## Discussion

The data presented here demonstrate that beta cell-specific PAK1 overexpression in HFD-induced glucose-intolerant mice suppressed disease progression despite the continued intake of a diabetogenic diet, coincident with reduced beta cell apoptosis, when compared with HFD-fed Ctrl mice. Interestingly, boosting PAK1 in the HFD-fed mouse islets significantly increased functional beta cell mass and islet insulin content. Collectively, these findings support the notion that elevating PAK1 in a beta cell-specific manner is sufficient to evoke systemic metabolic–homeostatic benefit via an in vivo mechanism involving increased insulin biogenesis and decreased beta cell apoptosis.

Mechanistically, we demonstrate that PAK1 can serve as a transcriptional modulator of *INS* gene expression. To the best of our knowledge, this is the first demonstration of PAK1 localising to the beta cell nucleus and its contribution to insulin biogenesis. Indeed, beta cell-specific PAK1 enrichment increases insulin content in type 2 diabetic human islets, while PAK1 deficit in β*Pak1*-iKO mouse islets results in reduced insulin granules. Our data show an increase in *INS*, *PDX1* and *NEUROD1* expression levels upon PAK1 supplementation in type 2 diabetic human islets. Transcriptomic data also suggest that beta cell-specific PAK1 induction modulates chromatin remodelling and organisation pathways. These data collectively imply a nuclear role for PAK1 in the beta cell. PAK1 has three nuclear localisation signals in the N-terminal domain that may facilitate its ability to localise in the nucleus, associate with chromatin and modulate the transcription [43]. PAK1 signalling has also been reported to regulate chromatin remodelling through the phosphorylation of histones and effector substrates and to modify their interactions with chromatin remodelling proteins [44, 45]. In addition, to direct PAK1-chromatin associations, PAK1 can also impact gene transcription through post-translational modification of transcriptional co-regulators such as C-terminal binding protein (CtBP), SMRT/HDAC1-associated repressor protein (SHARP) and Snail [46–48]. Further, PAK1 affects RNA polymerase II-dependent transcription through the Arp2/3 complex [49].

Our ChIP data using PDX1 or NEUROD1 antibodies in EndoC-βH1 cells overexpressing PAK1 suggests that PAK1 induces expression of these transcription factors in a glucose-responsive manner. Glucose is the central regulator of insulin gene expression and activates PAK1 [13, 50]. The regulation of insulin gene activation occurs via a complicated network involving nuclear factors besides PDX1, NEUROD1 and MafA [36, 51]. It is well established that MafA, PDX1 and NEUROD1 directly bind to the insulin promoter region and activate insulin transcription [9]. Indeed, PDX1 and NEUROD1 have a synergistic effect on insulin gene transcription and exist as a complex in beta cells [8, 9]. Our ChIP-seq analysis did not indicate direct PAK1 recruitment to the *INS*, *PDX1* or *NEUROD1* promoters, possibly suggesting that a bridging factor is involved in PAK1-induced expression of these genes. Importantly, beta cell identity genes are largely controlled by their enhancers [52]. Indeed, we observed that PAK1 was recruited to the enhancer regions of *INS*, *PDX1* and *NEUROD1*. Thus, enhancer modulation could be another potential mechanism by which PAK1 can activate insulin biogenesis.

The demonstration that PAK1 enrichment positively impacts insulin biogenesis in mice and in human type 2 diabetic islets adds to the existing list of positive PAK1 actions upon beta cell function, further expanding the potential for PAK1 as a therapeutic target. While this study was focused on deriving the molecular mechanisms underlying the observation of PAK1-induced insulin biogenesis, our RNA-seq results in PAK1-enriched ND and type 2 diabetic beta cells confirmed positive actions of PAK1 on beta cell function, via cytoskeletal dynamics (ESM. Fig. 7). Expanding the repertoire of PAK1 benefits to enhancement of *PDX1* and *NEUROD1* abundances, as well as their binding to *INS* promoter to activate *INS* gene transcription, provides the first molecular evidence for PAK1 in insulin content regulation. Given that insulin secretion and insulin biosynthesis are known to be kept in balance and that this study indicates PAK1 is a key regulator of this balance, therapeutically targeting PAK1 could provide access to the beta cell’s innate mechanisms for keeping both processes in balance.

## Supplementary Information

Below is the link to the electronic supplementary material.

ESM (PDF 3.39 MB)

## Data Availability

The raw and processed RNA-seq data and ChIP-seq data, which has been made publicly available at Gene Expression Omnibus (GEO) at https://www.ncbi.nlm.nih.gov/geo/, can be accessed in GSE239382.

## Abbreviations

AdRIP: Adenovirus rat insulin promoter
BCL2: BCL2 apoptosis regulator
CC3: Cleaved caspase 3
ChIP-seq: Chromatin immunoprecipitation sequencing
CPM: Counts per million
Ctrl: Control
DEG: Differentially expressed gene
Dox: Doxycycline
dTG: Double-transgenic mice
GLT: Glucolipotoxicity
GO: Gene ontology
GSEA: Gene set enrichment analysis
GSIS: Glucose-stimulated insulin secretion
HFD: High-fat diet
iβPAK1-Tg: Beta cell-specific PAK1 overexpressing transgenic mouse
IPA: Ingenuity pathway analysis
KEGG: Kyoto Encyclopedia of Gene and Genomes
KO: Knockout
LSD: Least significant difference
MafA: MAF bZIP transcription factor A
ND: Non-diabetic
NES: Normalised enrichment score
NEUROD1: Neuronal differentiation 1
PAK1: p21 (CDC42/RAC1) activated kinase 1
PDX1: Pancreatic and duodenal homeobox 1
βPAK1-iKO: Beta cell-specific *Pak1*-knockout mouse
qPCR: Quantitative PCR
RIP: Rat insulin promoter
sTg: Single-transgenic mice
TBP: TATA-box binding protein
TRE-h*PAK1*: Tet response element-h*Pak1* mouse
