# Supplementary material for: Beta cell-specific PAK1 enrichment ameliorates diet-induced glucose intolerance in mice by promoting insulin biogenesis and minimising beta cell apoptosis
Source: Diabetologia. 2024 Oct 15;68(1):152–65. doi: 10.1007/s00125-024-06286-2 (PMC11663170; doi:10.1007/s00125-024-06286-2)
Supplement: Supplementary file 1 — ESM (PDF 3.39 MB) [file 125_2024_6286_MOESM1_ESM.pdf]

Electronic supplementary material (ESM)

Beta cell-specific PAK1 enrichment ameliorates diet-induced glucose intolerance in mice by promoting insulin biogenesis and minimising beta cell apoptosis

Miwon Ahn[1], Sangeeta Dhawan[2], Erika M. McCown[1], Pablo A. Garcia[1], Supriyo Bhattacharya[3], Roland Stein[4] and Debbie C. Thurmond[1*]

[1]Arthur Riggs Diabetes and Metabolism Research Institute, Department of Molecular and Cellular Endocrinology, City of Hope, Duarte, CA, USA
[2]Arthur Riggs Diabetes and Metabolism Research Institute, Department of Translational Research and Cellular Therapeutics, City of Hope, Duarte, CA, USA
[3]Integrative Genomics Core, City of Hope, Duarte, CA, USA
[4]Department of Molecular Physiology and Biophysics, Vanderbilt University, Nashville, TN, USA

*Corresponding author
Debbie C. Thurmond, Ph.D. dthurmond@coh.org

Table of Contents

ESM Methods--S2-S7

ESM Table 1--S8

ESM Table 2--S9

ESM Table 3--S10-S16

ESM Table 4--S17-S25

ESM Table 5--S26

ESM Table 6--S27

ESM Table 7--S28

ESM Figure 1--S29

ESM Figure 2--S30

ESM Figure 3--S31

ESM Figure 4--S32

ESM Figure 5--S33

ESM Figure 6--S34

ESM Figure 7--S35

Human Islet Checklist

Supplementary Method

Human donor islets. Pancreatic human islets were obtained through the Integrated Islet Distribution Program (IIDP) and the City of Hope Islet Core (Duarte, CA, USA). Criteria for human donor islet acceptance are ≥ 80% purity and ≥ 75% viability. The islets were incubated in CMRL (Gibco, Grand Island, NY, USA) supplemented with 10% FBS and 1% penicillin/Streptomycin medium for 2 h to allow recovery from transport, and then handpicked using a green gelatine filter to eliminate residual non-islet material. Non-diabetic human islets (ND) without diabetes were treated with glucolipotoxic (GLT) mixture (16.7 mmol/l glucose plus 0.5 mmol/l palmitate; Sigma, St Louis, MO, USA) for 48 h. mRNA was quantified from islets by quantified real-time PCR. Islets from ND and type 2 diabetes human donors were transduced at 100 MOI with Ad-RIP-Ctrl or Ad-RIP-h*PAK1* for 1 h at 37°C. Transduced islets were then washed twice and incubated for 48 h in CMRL medium. Bulk RNA-seq was performed using isolated RNA from PAK1-transduced ND and T2D human islets. Insulin content was measured in 10 islets from type 2. Proteins and mRNA were extracted from T2D human islets transduced with PAK1, quantified by immunoblot and real-time PCR, respectively.

Transmission electron microscopy (TEM). Mouse pancreatic islets were isolated from 16-18-week-old male mice and immediately fixed with 2.5% glutaraldehyde, and 4% paraformaldehyde in 0.1 M cacodylate buffer (pH 7.3), for 2 h at room temperature. The specimens were stored at 4 ^{o}C and later post-fixed for 1 h in 0.1 M cacodylate buffer, 1% OsO_4. After rinsing in distilled water, specimens were *en bloc* stained for 1 h in 1% uranyl acetate in distilled water. Specimens were serial dehydrated with ethanol and embedded in Eponate. Ultrathin (70-nm) sections were acquired by Leica Ultracut, picked up on copper grids, post-stained with lead citrate and viewed with a FEI Tecnai T12 operating at 120 KV.

INS1 832/13 cell culture: INS1 832/13 cells were cultured in RPMI 1640 medium supplemented with 10 mol/l HEPES, 10% FBS, 0.5% penicillin/Streptomycin, 2 mmol/l L-glutamine, 1 mmol/l sodium pyruvate, and 50 μmol/l β-mercaptoethanol.

Adenovirus. To produce CMV-driven adenovirus, the cDNA of human PAK1 tagged with myc was subcloned into the AdCMV shuttle vector. Adenovirus rat insulin promoter (RIP)-driven h*PAK1* was generated as described

previously [2]. The empty AdCMV vector and AdRIP-gfp were used as the control. Viraquest (North Liberty, IA, USA) generated all the adenoviruses.

PAK1 knockdown using siRNA. Both INS1 832/13 cells and human EndoC-βH1 cells were transfected with either siPAK1 (#S103082926 for INS1 832/13 or #S100605696 for human EndoC-βH1 from Qiagen, Valencia, CA, USA) or siCon (#1027310 from Qiagen, Valencia, CA, USA) using RNAiMAX (Invitrogen, Carlsbad, CA, USA) for 48 h. Subsequently, Insulin content was measured.

Insulin content: To quantify the insulin content of mouse islets and clonal beta cell lines, the protein lysate was utilized for insulin ELISA. The ALPCO ultrasensitive ELISA kit for mouse islet, the ALPCO rat insulin ELISA Kit for INS1 832-13 (ALPCO, Salem, NH, USA), and Mercodia human insulin ELISA kit for human EndoC-βH1 (Mercodia, Uppsala, Sweden) were used according to manufacturer's protocol. For human islets,10 islets from each group were lysed for insulin content analysis using the ultrasensitive human insulin RIA kit (Millipore, MA, USA).

ND human islet perifusion. Thirty transduced islets were handpicked onto a column for perifusion analysis [1]. Control islets were run in parallel columns with experimental (PAK1 transduced) islets. Islets were then perifused at a flow rate of 0.3 ml/min for 10 min in Krebs-Ringer bicarbonate buffer (KRBB; 10 mmol/l HEPES (pH7.4), 134 nmol/l NaCl, 5 mmol/l $NaHCO_3$, 4.8 mmol/l KCl, 1 mmol/l $CaCl_2$, 1.2 mmol/l $MgSO_4$, 1.2 mmol/l KH_2PO_4 and 0.05% BSA) containing 2.8 mmol/l glucose with eluted fractions captured at 1 min intervals, followed by glucose stimulation of 16.7 mmol/l glucose for 35 min. Insulin secreted into eluted fractions was quantitated by the ultra-sensitive human insulin RIA kit (Millipore, Billerica, CA, USA).

QPCR. RNA was isolated using RNeasy Plus Mini Kit (Qiagen, Valencia, CA, USA) following the manufacturer's instructions. Real-time PCR was performed using the QuantiTect SYBR Green one-step qRT-PCR kit (Qiagen, Valencia, CA, USA) with 50 ng of RNA. The QuantStudio 3 (Thermo Fisher Scientific, Rockford, IL, USA) was used to run 40 cycles of real-time PCR. For cDNA synthesis, the reaction was carried out at 50 °C for 30 min

and 95 °C for 2 min. Each cycle consisted of running the reaction at 95 °C for 15 s, 58 °C for 30 sec and 72 °C for 30 s.

Immunoblotting: Transduced primary human islets (n=30), or mouse islets (n=30) were harvested using 1% Nonidet P-40 lysis buffer (1% NP-40, 25 mmol/l HEPES pH 7.4, 10% glycerol, 50 µmol/l sodium fluoride, 10 mmol/l sodium pyrophosphate, 1 mmol/l sodium vanadate, 137 mmol/l sodium chloride, 1 mmol/l phenylmethylsulphonyl fluoride, proteases and Phosphatase inhibitor (Thermo Fisher Scientific, Rockford, IL, USA). Transiently transfected human EndoC-βH1 cells or INS1 832/13 cells were separated into nucleus and non-nuclear fractions using NE-PER kit (Thermo Fisher Scientific, Rockford, IL, USA). Proteins were resolved by SDS-PAGE and transferred to polyvinylidene fluoride membranes (PVDF) for immunoblotting using 1: 1000 dilutions of rabbit anti-PAK1, rabbit anti-TBP, rabbit anti-cleaved caspase 3, PDX1 and NEUROD1 (Cell Signalling, Danvers, MA, USA), rabbit-anti-BCL2 (Millipore, MA, USA), mouse anti-myc (Abcam, Cambridge, MA, USA), and 1: 5,000 dilution of mouse anti-tubulin (Sigma, St. Louis, MO, USA). Secondary goat anti-mouse (1:20,000) and anti-rabbit horseradish peroxidase (1:10,000) were used (Bio-Rad, Hercules, CA, USA). The enhanced chemiluminescence (ECL) kit and ECL prime kit were purchased from Amersham Biosciences (Pittsburgh, PA, USA). Immunoreactive bands were imaged with a Chemi-Doc Touch (Bio-Rad, Hercules, CA, USA).

RNA-seq sample preparation: To extract RNA from transduced PAK1 or control human islets, we used the Qiagen RNeasy Plus Mini Kit. RNA QC was done with RNA 6000 nano kit (Agilent, Cat #5076-1511, Valencia, CA, USA) for bulk RNA-seq. We used RNA that RNA integrity number (RIN) values for all samples were > 7.0. According to the user manual, 250 ng of total RNA from each sample was used for sequencing library preparation with KAPA mRNA HyperPlus Kit (KAPA Biosystems, Cat # KK8514, Wilmington, MA, USA). The library QC was checked with an Agilent bioanalyzer DNA high-sensitivity chip (Agilent, Cat#5067-4626, Santa Clara, CA, USA) and Qubit dsDNA High Sensitivity kit (Themo Fisher, Cat # Q33231, Rockford, IL, USA) was used for library quantification. The final libraries were sequenced on Hiseq2500 (Illumina, San Diego, CA, USA) with a single read mode of 51 cycles, plus 7 cycles of index sequencing with V4 kit.

ChIP-seq and analysis: Genomic DNA (Input) was prepared by treating aliquots of chromatin with RNase (#R5250, Sigma St. Louis, MO, USA), proteinase K (#P6556, Sigma St. Louis, MO, USA) and heat (overnight, 65 °C) for de-crosslinking, followed by SPRI beads clean up (#B23318, Beckman Coulter, Brea, CA, USA) and quantified using a CLARIOstar microplate reader (BMG Labtech, San Jose, CA, USA). Extrapolation to the original chromatin volume allowed quantitation of the total chromatin yield. An aliquot of chromatin (15 μg) was precleared with protein agarose beads (#15918014, Invitrogen, Waltham, MA, USA). Genomic DNA regions of interest were isolated using 5 μl antibody against Myc-tag (#ab9132, Abcam, Cambridge, MA, USA). Complexes were washed, eluted from the beads with SDS buffer, and subjected to RNase and proteinase K treatment. Crosslinks were reversed by incubation overnight at 65 °C, and ChIP DNA was purified by phenol-chloroform extraction and ethanol precipitation. Illumina sequencing libraries were prepared from the ChIP and Input DNAs using the standard consecutive enzymatic steps of end-polishing, dA-addition, and adaptor ligation using an automated system (Apollo 342, Wafergen Biosystems/Takara). The 75-nt single-end (SE75) sequence reads generated by Illumina sequencing (using NextSeq 500) were mapped to the genome using the BWA algorithm ("bwa aln/samse" with default settings) [3]. Only reads that passed Illumina's purity filter, aligned with no more than 2 mismatches, and mapped uniquely to the genome were used in the subsequent analysis. In addition, duplicate reads ("PCR duplicates") were removed for further analysis. Pathway enrichment analysis was conducted using the QIAGEN Ingenuity Pathway Analysis (IPA) software [4] and the topGO R package [5]. Enriched DNA sequence motifs were identified using Hypergeometric Optimization of Motif EnRichment (HOMER) [6].

ChIP Assay. Transduced myc-hPAK1 or control EndoC-βH1 cells were incubated in low glucose (2.8 mmol/l) media overnight, then pre-incubated in KRBB containing 2.8 mmol/l glucose for 1 h. Subsequently, the buffer was changed to either 2.8 mmol/l or 16.7 mmol/l glucose and incubated for 30 min. Cells were treated with 1% paraformaldehyde at room temperature for 20 min to crosslink the DNA and proteins in the chromatin. Formaldehyde was quenched by adding glycine to a final concentration of 125 mmol/l, followed by cell lysis in a buffer consisting of 50 mmol/l Tris HCl pH 8.0, 10 mmol/l EDTA, and 1% SDS, supplemented with 1X complete proteinase inhibitors (EMD Biosciences, Millipore, USA). The lysate was sonicated to generate DNA fragments with an average size of 500 bp using Bioruptor (Diagenode, Denville, NJ, USA). A total of 2.5 μg of antibody was

bound to 20 μl Protein-A/G Dynabeads (Invitrogen, Waltham, MA, USA), depending on the antibody isotype, for 2 h at 4°C with agitation. The following antibodies were used: PDX1 (goat, kind gift from Dr. Chris Wright, Vanderbilt University, TN, USA), NEUROD1 (Thermo Fisher, rabbit monoclonal IgG MA5-32626, Rockford, IL, USA), control goat IgG (Thermo Fisher, 02-6202, Rockford, IL, USA) and control rabbit IgG (Diagenode, KCH-504-250, Denville, NJ, USA). Beads carrying specific antibodies or respective IgG controls were incubated with chromatin for 2 h at 4°C with agitation, after reserving a fraction of the chromatin as input. The bead-chromatin complexes were captured using a magnetic stand, and washed 3X with RIPA buffer containing proteinase inhibitors, followed by DNA elution, un-crosslinking, and precipitation. After elution, real-time PCR were performed to amplify the DNA fragment containing the insulin promoter region covering the PDX1 and NEUROD1 binding sites with primers in ESM Table 2.

Glucose tolerance test (IPGTT) and insulin tolerance tests (IPITT). For IPGTT, blood samples were collected from the tail vein of 6 h fasted mice at 0, 15, 30, 60, 90, and 120 min after intraperitoneal injection of glucose (2 g/kg body weight). For IPITT, 6-h fasted mice were intraperitoneally injected with Humulin R (0.75 units/kg, Eli Lilly, Inc. Indianapolis, IN, USA); tail vein blood samples were collected at 0, 15, 30, 45, 60, and 90 min after injection. Glucose levels were measured using HemoCue glucometers (Brea, CA, USA).

Mouse islet static culture. Pancreatic mouse islets were isolated, as previously described [7]. Ten hand-picked islets were incubated in KRBB containing 2.8 mmol/l glucose for 1 h. Then, the buffer was changed to either 2.8 or 16.7 mmol/l glucose and incubated for another 1 h. The supernatant was collected for quantification of insulin release using the insulin ELISA (ALPCO, Salem, NH, USA). Islets were subsequently lysed with 1% NP-40 lysis buffer for quantitation of total cellular protein and insulin content.

TUNEL Assay. Fluorescence immunostaining was performed on pancreatic sections to detect insulin, using guinea pig anti-insulin (1:100; Abcam, Cambridge, MA, USA) and Alexa Fluor 488 goat anti-guinea pig (1:100; Invitrogen, Carlsbad, CA, USA). Apoptosis cells were detected using the TUNEL in Situ TMR-Red Cell Death Detection Kit (Sigma-Aldrich, St. Louis, MO, USA). The sections were scanned using a Keyence microscope

(Keyence Itasca, IL, USA), and the results were presented, indicating the percentage of TUNEL+ cells relative to the total number of insulin+ beta cells.

References

[1] Wang Z, Oh E, Thurmond DC (2007) Glucose-stimulated Cdc42 signaling is essential for the second phase of insulin secretion. J Biol Chem 282(13): 9536-9546

[2] Ahn M, Yoder SM, Wang Z, et al. (2016) The p21-activated kinase (PAK1) is involved in diet-induced beta cell mass expansion and survival in mice and human islets. Diabetologia 59(10): 2145-2155. 10.1007/s00125-016-4042-0

[3] Li H (2013) Aligning sequence reads, clone sequences and assembly contigs with BWA-MEM. arXiv preprint arXiv:13033997

[4] Kramer A, Green J, Pollard J, Jr., Tugendreich S (2014) Causal analysis approaches in Ingenuity Pathway Analysis. Bioinformatics 30(4): 523-530. 10.1093/bioinformatics/btt703

[5] Rahnenfuhrer AAJ (2022) topGO: Enrichment Analysis for Gene Ontology. R package version 2.48.0. In:

[6] Heinz S, Benner C, Spann N, et al. (2010) Simple combinations of lineage-determining transcription factors prime cis-regulatory elements required for macrophage and B cell identities. Mol Cell 38(4): 576-589. 10.1016/j.molcel.2010.05.004

[7] Spurlin BA, Thurmond DC (2006) Syntaxin 4 Facilitates Biphasic Glucose-Stimulated Insulin Secretion from Pancreatic Beta Cells. Mol Endocrinol 20(1): 183-193

ESM Table 1. Human islet donor information

RRID/ COH	SEX	Age (year)	BMI	HbA1c	Race or Ethnicity	Diabetes	Experimental use of islets
COH-Hu-1013	M	35	27.4	5.4	White	Non-Diabetes	RNA-seq
COH-Hu-1031	M	37	30.0	5.3	Asian	Non-Diabetes	RNA-seq
COH-Hu-1107	M	45	26.0	4.6	Hispanic or Latino	Non-Diabetes	RNA-seq
SAMN08768767	M	35	31.5	4.9	White	Non-Diabetes	RNA-seq
COH-Hu-1022	M	68	31.0	7.9	Hispanic or Latino	T2D	RNA-seq
SAMN08768773	M	51	35.6	7.1	Hispanic or Latino	T2D	RNA-seq
SAMN08768784	F	52	42.8	6.6	Hispanic or Latino	T2D	RNA-seq
SAMN08774476	F	39	23.5	6.0	Hispanic or Latino	Non-Diabetes	Perifusion
SAMN08774821	M	23	32.9	4.7	Hispanic or Latino	Non-Diabetes	Perifusion
SAMN08774955	M	48	30.7	5.8	Hispanic or Latino	Non-Diabetes	Perifusion
SAMN28156887	M	29	22.2	5.6	Hispanic or Latino	Non-Diabetes	Perifusion
SAMN10832912	M	54	33.7	8	Black/Afr. Am.	T2D	RT-PCR, Protein
SAMN10953189	F	56	23.1	7.2	White	T2D	RT-PCR, Protein
SAMN17833574	M	45	35.6	5.0	Black/Afr. Am.	T2D	RT-PCR, Protein
SAMN25980818	M	55	31	6.8	Asian	T2D	RT-PCR, Protein
SAMN19233334	F	47	35.1	6.4	Hispanic or Latino	T2D	RT-PCR
SAMN21845647	M	62	34.9	6.8	White	T2D	RT-PCR
COH-Hu1000	M	49	28.9	5.1	Hispanic or Latino	Non-Diabetes	GLT, 48 h
SAMN08774955	M	48	30.7	5.8	White	Non-Diabetes	GLT, 48 h
SAMN08774960	F	48	32.8	5.0	White	Non-Diabetes	GLT, 48 h
SAMN08774965	M	52	29.0	NA	White	Non-Diabetes	GLT, 48 h
Hu1288	M	58	38.4	6.2	Hispanic or Latino	T2D	Protein
Hu1277	M	63	25.2	10.7	White	T2D	Protein
SAMN39243818	M	62	43	6.1	White	T2D	Protein
SAMN37284997	F	62	43	6.1	Black/Afr. Am.	T2D	Protein
Hu1293	M	43	29.9	6.7	White	T2D	Protein
SAMN40498032	F	56	33.4	6.7	White	T2D	Protein

RRID, Research Resource Identifier; *BMI*, body mass index; *HbA1c*, glycated haemoglobin; *Afr. Am.*; African Americans;*T2D*, type 2 diabetes; COH, City of Hope islet core; NA, Not Applicable

ESM Table 2. PCR Primers

Name	Sequence	
h-PAK1	5’-GGAGTTTACGGGAATGCCAGAG-3’<br>5’-CAGCCTGCGGGTTTTTCTTC-3’	RT-PCR
h-Insulin	5’-CTCACACCTGGTGGAAGCTC-3’<br>5’-AGAGGGAGCAGATGCTGGTA-3’	RT-PCR
h-NeuroD1	5’-AAGCCATGAACGCAGAGGAG-3’<br>5’-CTGAACGAAGGAGACCAGGT-3’	RT-PCR
h-Pdx1	5’-CCTTTCCCATGGATGAAGTC-3’<br>5’-TTCAACATGACAGCCAGCTC-3’	RT-PCR
h-MAFA	5’-CGCCAGCTTCTCGTATTTCT-3’<br>5’-TTCAGCAAGGAGGAGGTCAT-3’	RT-PCR
h-MAFB	5’-GCCAAACCGCATAGAGAAC-3’<br>5’-GGGGATAAGGGAAGGAAAG-3’	RT-PCR
h-tubulin	QIAGEN Cat#QT00089775	RT-PCR
h-Insulin promoter	5’-TCCGGAAATTGCAGCCTCAG-3’<br>5’-TTCTGATGCAGCCTGTCCTG-3’	ChIP Assay

<u>ESM Table 3.</u> DEG list for bulk RNA-seq in T2D human islets

	LogFC	AveExpr	t	P.Value
**PAK1**	3.467	6.895	11.439	1.03E-07
**MMP3**	1.370	0.530	6.491	3.32E-05
**LBP**	0.948	-0.443	4.861	4.18E-04
**PI3**	1.661	0.153	4.676	5.70E-04
**NTNG2**	0.701	1.319	4.396	9.20E-04
**SOX10**	1.306	-1.060	4.371	9.62E-04
**PI4KAP1**	1.361	-1.562	4.299	1.09E-03
**MT2A**	0.661	2.586	4.228	1.23E-03
**MUC4**	1.390	-0.823	4.180	1.34E-03
**CD93**	-0.543	5.313	-3.890	2.24E-03
**RGS5**	-0.480	6.156	-3.694	3.19E-03
**FBN1**	-0.459	6.385	-3.680	3.27E-03
**PLEKHG1**	-0.479	2.857	-3.585	3.88E-03
**GABRP**	0.974	-0.076	3.493	4.59E-03
**MIR210HG**	0.601	1.989	3.489	4.62E-03
**COL12A1**	-0.426	6.248	-3.449	4.97E-03
**SLC7A11**	-0.511	4.438	-3.424	5.20E-03
**ADGRL4**	-0.604	3.225	-3.415	5.29E-03
**MYLK**	-0.469	4.679	-3.404	5.40E-03
**COL11A1**	-0.681	1.072	-3.377	5.67E-03
**GABRE**	1.079	-0.826	3.363	5.82E-03
**NPR3.00**	-0.641	1.219	-3.302	6.50E-03
**DUOXA2**	0.485	5.625	3.288	6.67E-03
**CHSY3**	-0.656	0.818	-3.267	6.94E-03
**SPAG4**	0.490	1.695	3.256	7.07E-03
**GJC1**	-0.489	2.260	-3.256	7.08E-03
**PAEP**	0.798	-1.194	3.217	7.59E-03
**PRKG1**	-0.495	2.847	-3.194	7.93E-03
**CRHR1**	0.717	-1.204	3.139	8.78E-03
**PTPRB**	-0.449	2.760	-3.138	8.78E-03
**SLPI**	0.576	2.816	3.125	9.01E-03
**KDR**	-0.502	4.206	-3.095	9.51E-03
**SOCS1**	0.452	2.911	3.074	9.88E-03
**VIP**	1.413	-3.698	3.072	9.92E-03
**CBLN3**	0.591	0.405	3.069	9.98E-03
**WNT5A**	-0.399	5.829	-3.058	1.02E-02
**PIEZO2**	-0.510	2.874	-3.044	1.04E-02
**MIF-AS1**	0.654	1.488	3.034	1.06E-02
**FAM198B**	-0.470	4.570	-3.033	1.07E-02
**EBF1**	-0.455	2.598	-3.025	1.08E-02
**HEG1**	-0.433	4.967	-3.002	1.13E-02
**ATHL1**	0.800	1.145	2.992	1.15E-02
**COL4A1**	-0.451	8.793	-2.978	1.18E-02
**LY6G5B**	0.966	-0.825	2.969	1.20E-02
**PPFIA4**	1.060	-0.691	2.935	1.28E-02
**LEFTY2**	-0.783	-1.499	-2.935	1.28E-02
**ECEL1**	0.386	4.008	2.934	1.28E-02
**RPS29**	0.574	5.456	2.933	1.28E-02
**GSDMB**	0.671	-0.150	2.923	1.31E-02

**PTPN14**	-0.383	3.393	-2.896	1.37E-02
**NCCRP1**	0.844	-0.166	2.885	1.40E-02
**FCHSD1**	0.475	2.508	2.878	1.42E-02
**KCNK12**	0.882	-1.157	2.854	1.48E-02
**CEACAM5**	0.968	-0.797	2.846	1.50E-02
**EYA4**	-0.778	-0.515	-2.843	1.51E-02
**ITPRIPL2**	-0.354	6.190	-2.843	1.51E-02
**WAS**	0.698	-0.786	2.823	1.57E-02
**COL5A2**	-0.353	7.692	-2.821	1.57E-02
**AHNAK**	-0.371	6.463	-2.817	1.59E-02
**RBMS3**	-0.487	1.830	-2.815	1.59E-02
**VCAN**	-0.356	7.689	-2.799	1.64E-02
**CYP1B1**	-0.354	5.226	-2.793	1.66E-02
**CEBPD**	0.344	3.976	2.788	1.67E-02
**XYLT1**	-0.358	3.966	-2.786	1.68E-02
**CRCT1**	0.531	0.162	2.776	1.71E-02
**LRRC8C**	-0.447	3.247	-2.769	1.73E-02
**NOL3**	0.367	4.060	2.751	1.79E-02
**ADAM12**	-0.341	4.682	-2.751	1.79E-02
**KITLG**	-0.485	4.427	-2.748	1.80E-02
**CLIC4**	-0.447	6.681	-2.736	1.84E-02
**DOK6**	-0.441	2.431	-2.734	1.85E-02
**SERPINB7**	1.079	2.180	2.733	1.85E-02
**FXYD2**	0.517	0.389	2.724	1.88E-02
**MYCT1**	-0.473	1.786	-2.717	1.91E-02
**CA9**	0.472	2.827	2.715	1.91E-02
**ALDH3B2**	0.708	-2.596	2.705	1.95E-02
**PAPPA**	-0.352	3.934	-2.704	1.95E-02
**CHSY1**	-0.330	5.109	-2.701	1.96E-02
**ADAMTS12**	-0.353	4.837	-2.697	1.98E-02
**RGL3**	0.595	2.923	2.696	1.98E-02
**GAN**	-0.437	2.145	-2.694	1.99E-02
**LPP**	-0.359	6.711	-2.688	2.01E-02
**SNHG7**	0.358	3.486	2.680	2.04E-02
**FLT1**	-0.386	5.714	-2.670	2.08E-02
**NAALADL2**	-0.435	2.063	-2.659	2.12E-02
**ARMCX4**	-0.356	4.306	-2.658	2.12E-02
**KLHDC7B**	0.658	-1.510	2.654	2.14E-02
**PTPRG**	-0.340	4.212	-2.653	2.14E-02
**MUC6**	0.409	1.682	2.639	2.20E-02
**MYO15B**	0.839	0.951	2.628	2.24E-02
**GPC6**	-0.366	6.004	-2.621	2.27E-02
**ACHE**	0.389	2.326	2.618	2.29E-02
**ESM1**	-0.336	4.413	-2.617	2.29E-02
**IL17RB**	0.414	2.467	2.597	2.37E-02
**TMTC1**	-0.501	1.031	-2.596	2.38E-02
**CXCL10**	-1.229	-0.037	-2.596	2.38E-02
**LOC100996634**	1.148	-1.512	2.595	2.38E-02
**FGB**	0.366	3.473	2.587	2.42E-02
**LTBP1**	-0.322	5.793	-2.582	2.44E-02
**DOCK11**	-0.408	2.010	-2.578	2.46E-02
**CDH17**	-0.645	0.488	-2.576	2.46E-02

**SECTM1**	0.421	1.011	2.574	2.47E-02
**GPR161**	-0.359	2.454	-2.564	2.52E-02
**ITPR1**	-0.322	5.466	-2.564	2.52E-02
**SLC1A2**	-0.475	1.595	-2.564	2.52E-02
**RPL14**	0.382	6.151	2.559	2.54E-02
**GUSBP11**	0.746	1.942	2.558	2.55E-02
**BCAT1**	-0.537	5.178	-2.557	2.55E-02
**ITGA1**	-0.323	7.221	-2.557	2.55E-02
**COL6A3**	-0.366	9.872	-2.556	2.56E-02
**ITPR2**	-0.350	5.400	-2.556	2.56E-02
**ITGB3**	-0.313	4.593	-2.555	2.56E-02
**AMOTL1**	-0.316	5.239	-2.549	2.59E-02
**DLC1**	-0.328	4.092	-2.543	2.62E-02
**BCL2**	-0.392	1.917	-2.542	2.62E-02
**PRKDC**	-0.317	7.133	-2.541	2.63E-02
**NPEPL1**	0.363	3.421	2.540	2.63E-02
**SHCBP1**	-0.522	0.602	-2.536	2.65E-02
**NTRK2**	-0.316	4.833	-2.536	2.65E-02
**CDH11**	-0.307	5.644	-2.535	2.66E-02
**RUNX1**	-0.308	5.951	-2.534	2.66E-02
**ADGRF5**	-0.345	4.200	-2.534	2.66E-02
**IRF7**	0.447	1.151	2.534	2.66E-02
**PRR5**	0.611	-0.275	2.533	2.67E-02
**DYSF**	-0.456	2.167	-2.532	2.67E-02
**FGF13**	-0.543	0.271	-2.530	2.68E-02
**FN1**	-0.372	11.083	-2.527	2.69E-02
**GADD45G**	0.349	3.379	2.526	2.70E-02
**CENPF**	-0.397	1.946	-2.524	2.71E-02
**ADHFE1**	0.579	0.264	2.523	2.72E-02
**KLHL17**	0.717	1.054	2.517	2.75E-02
**CBL**	-0.318	4.706	-2.511	2.78E-02
**ATP11C**	-0.397	2.801	-2.508	2.79E-02
**ZFPM2**	-0.451	1.141	-2.507	2.79E-02
**ZNF692**	0.744	1.445	2.507	2.80E-02
**BMPR2**	-0.357	7.088	-2.499	2.84E-02
**LAMA2**	-0.314	4.594	-2.496	2.85E-02
**KIAA0922**	-0.329	3.619	-2.490	2.88E-02
**MICAL1**	0.348	3.558	2.489	2.89E-02
**KCNC3**	0.416	2.930	2.489	2.89E-02
**GBP4**	-0.446	2.115	-2.488	2.90E-02
**LHFPL2**	-0.300	5.312	-2.486	2.90E-02
**FNDC1**	-0.628	2.154	-2.484	2.92E-02
**RAD51**	-0.630	-1.062	-2.483	2.92E-02
**PRLR**	-0.373	5.320	-2.481	2.93E-02
**ADCY10P1**	0.962	-1.409	2.477	2.96E-02
**MRVI1**	-0.426	1.723	-2.476	2.96E-02
**INS**	0.399	11.050	2.474	2.97E-02
**ASCL1**	0.428	1.440	2.472	2.98E-02
**EPPK1**	-0.399	1.335	-2.470	2.99E-02
**PODXL**	-0.351	5.214	-2.470	2.99E-02
**PRSS22**	0.377	2.656	2.469	3.00E-02
**NOTCH2**	-0.316	6.119	-2.468	3.00E-02

**GRIK3**	0.410	1.766	2.466	3.01E-02
**MEF2C**	-0.336	3.330	-2.465	3.02E-02
**VWF**	-0.431	4.149	-2.463	3.03E-02
**ADAMTS5**	-0.384	2.800	-2.438	3.17E-02
**PDK3**	-0.301	6.938	-2.434	3.19E-02
**LENG8**	0.655	5.025	2.431	3.21E-02
**PLEKHG5**	0.413	1.370	2.431	3.21E-02
**SHE**	-0.514	0.344	-2.426	3.24E-02
**RGS11**	0.761	1.637	2.426	3.24E-02
**COL10A1**	-0.322	3.110	-2.424	3.25E-02
**MFI2-AS1**	0.682	-0.951	2.422	3.26E-02
**ATP8A1**	-0.351	5.806	-2.421	3.27E-02
**HILPDA**	0.365	3.456	2.419	3.28E-02
**ZNF704**	-0.365	3.664	-2.418	3.29E-02
**C9orf142**	0.479	1.813	2.418	3.29E-02
**CALD1**	-0.309	7.791	-2.418	3.29E-02
**ZNF697**	-0.308	4.291	-2.414	3.31E-02
**RPL31P11**	0.347	2.511	2.410	3.34E-02
**KIRREL**	-0.315	3.389	-2.407	3.35E-02
**PRDM1**	-0.330	3.510	-2.405	3.37E-02
**LIFR**	-0.416	2.995	-2.398	3.41E-02
**DYNC2H1**	-0.311	4.842	-2.398	3.41E-02
**MIRLET7BHG**	0.872	-0.218	2.395	3.42E-02
**TMPRSS3**	0.479	0.243	2.395	3.43E-02
**MIF**	0.393	6.465	2.393	3.44E-02
**SEMA5A**	-0.307	5.861	-2.393	3.44E-02
**MLXIPL**	0.539	4.674	2.392	3.45E-02
**RPS14P3**	0.311	4.031	2.388	3.47E-02
**COL27A1**	0.547	4.089	2.387	3.47E-02
**CHRDL2**	0.479	-1.128	2.386	3.49E-02
**KIAA1462**	-0.301	4.341	-2.385	3.49E-02
**HSF4**	0.930	1.561	2.384	3.49E-02
**C10orf128**	0.585	-0.532	2.381	3.51E-02
**PURB**	-0.367	5.920	-2.381	3.51E-02
**SLC18A3**	0.625	-2.129	2.379	3.53E-02
**DSEL**	-0.344	2.928	-2.379	3.53E-02
**TMEM92**	0.454	-0.163	2.377	3.54E-02
**MUTYH**	0.455	1.280	2.375	3.55E-02
**SLC9A6**	-0.316	4.800	-2.375	3.55E-02
**A2M**	-0.335	6.407	-2.374	3.56E-02
**NCKAP5**	0.337	2.529	2.372	3.58E-02
**LRP1**	-0.351	6.821	-2.371	3.58E-02
**SNHG12**	0.751	0.865	2.364	3.62E-02
**SLC25A25-AS1**	0.556	1.093	2.364	3.63E-02
**SLC5A3**	-0.444	4.213	-2.364	3.63E-02
**EPT1**	-0.332	6.082	-2.361	3.64E-02
**CABP7**	0.326	5.672	2.359	3.65E-02
**PEAK1**	-0.294	5.221	-2.357	3.67E-02
**CRIM1**	-0.299	5.213	-2.354	3.69E-02
**F2R**	-0.286	5.353	-2.352	3.70E-02
**ANKRD50**	-0.337	4.654	-2.352	3.71E-02
**ZXDB**	-0.355	3.758	-2.351	3.71E-02

**CYP4F24P**	1.033	0.697	2.350	3.72E-02
**GNB4**	-0.371	4.304	-2.347	3.73E-02
**ST6GAL2**	-0.448	1.049	-2.346	3.74E-02
**LMNTD2**	0.744	-0.734	2.346	3.75E-02
**UCA1**	0.436	0.489	2.345	3.75E-02
**PABPC1L**	0.877	-0.018	2.337	3.81E-02
**MIB1**	-0.441	6.010	-2.336	3.81E-02
**ITGA4**	-0.322	3.217	-2.335	3.82E-02
**SERINC5**	-0.284	5.381	-2.333	3.83E-02
**PLEKHG4**	0.554	0.608	2.329	3.86E-02
**WDR38**	0.643	-0.502	2.328	3.87E-02
**HNF1A-AS1**	0.673	0.069	2.328	3.87E-02
**FAT1**	-0.296	7.377	-2.327	3.87E-02
**DXO**	0.349	2.919	2.327	3.88E-02
**DMBT1**	0.507	1.316	2.326	3.88E-02
**KPTN**	0.446	1.062	2.319	3.93E-02
**FKBP1AP1**	-0.411	0.983	-2.317	3.94E-02
**SYCN**	0.431	-0.336	2.316	3.95E-02
**STON1**	-0.391	2.172	-2.316	3.95E-02
**CHN2**	-0.396	1.872	-2.313	3.97E-02
**POLD4**	0.295	3.778	2.312	3.98E-02
**CBLN1**	0.406	0.800	2.310	4.00E-02
**CCDC159**	0.384	1.933	2.309	4.00E-02
**PSAT1**	-0.348	3.331	-2.308	4.01E-02
**SNRNP70**	0.310	5.721	2.308	4.01E-02
**HEXDC**	0.421	1.944	2.308	4.01E-02
**SHROOM4**	-0.369	1.689	-2.307	4.01E-02
**TCF21**	-0.473	0.599	-2.307	4.02E-02
**CAPN10**	0.372	2.156	2.304	4.04E-02
**NORAD**	-0.330	7.970	-2.304	4.04E-02
**RRAS**	0.309	2.935	2.303	4.05E-02
**ZAK**	-0.302	4.464	-2.301	4.06E-02
**GIPR**	0.712	2.295	2.300	4.06E-02
**CCDC130**	0.328	2.853	2.296	4.10E-02
**SULF1**	-0.287	6.863	-2.295	4.10E-02
**KIAA1549L**	-0.336	4.556	-2.295	4.11E-02
**SLC26A2**	-0.328	4.950	-2.291	4.14E-02
**CCDC183**	0.703	0.107	2.289	4.15E-02
**SV2B**	-0.316	3.453	-2.289	4.15E-02
**PRRG1**	-0.370	3.028	-2.287	4.16E-02
**KRT19**	0.308	3.387	2.286	4.17E-02
**C3orf80**	-0.373	3.415	-2.285	4.18E-02
**C3orf36**	-0.537	-1.140	-2.285	4.18E-02
**SAA1**	0.654	-1.117	2.284	4.18E-02
**EDNRA**	-0.294	5.278	-2.284	4.19E-02
**ENTHD2**	0.517	1.374	2.284	4.19E-02
**NPY1R**	-0.482	1.258	-2.283	4.19E-02
**CNOT6L**	-0.360	5.323	-2.282	4.20E-02
**POSTN**	-0.290	6.209	-2.281	4.21E-02
**KLB**	-0.354	3.550	-2.280	4.22E-02
**MKI67**	-0.321	2.629	-2.279	4.22E-02
**MS4A6A**	-0.607	-0.419	-2.279	4.22E-02

**HEY2**	-0.501	1.021	-2.275	4.26E-02
**FAM117B**	-0.356	3.698	-2.271	4.29E-02
**PXDN**	-0.331	6.991	-2.270	4.29E-02
**IFFO1**	0.449	2.397	2.270	4.29E-02
**FAM193B**	0.488	2.962	2.257	4.39E-02
**MT1X**	0.329	2.249	2.257	4.40E-02
**MIR1282**	0.569	-0.152	2.248	4.46E-02
**PGR**	-0.281	5.591	-2.246	4.48E-02
**LTBP2**	-0.341	5.421	-2.246	4.48E-02
**SSR1**	-0.293	8.094	-2.244	4.50E-02
**LPIN3**	0.560	2.434	2.240	4.53E-02
**TXNDC5**	0.603	-0.840	2.239	4.54E-02
**COL4A2**	-0.370	8.654	-2.238	4.54E-02
**CHST8**	0.324	3.075	2.238	4.55E-02
**PWAR5**	-0.513	3.207	-2.237	4.55E-02
**SACS**	-0.334	4.858	-2.237	4.56E-02
**IPCEF1**	-0.509	-0.244	-2.233	4.58E-02
**SGCD**	-0.369	3.220	-2.233	4.59E-02
**ADM**	0.370	4.168	2.232	4.59E-02
**WDR90**	0.557	0.890	2.229	4.62E-02
**SOWAHC**	-0.349	3.866	-2.228	4.62E-02
**ACAP3**	0.331	4.265	2.228	4.63E-02
**PCAT6**	0.551	-0.576	2.226	4.65E-02
**FGL2**	-0.381	2.114	-2.222	4.68E-02
**CBX5**	-0.276	7.562	-2.221	4.68E-02
**ODF3B**	0.400	0.601	2.219	4.70E-02
**HECA**	-0.348	4.435	-2.219	4.70E-02
**DOK3**	0.647	-0.207	2.219	4.71E-02
**DACT1**	-0.280	4.065	-2.218	4.71E-02
**PIDD1**	0.603	0.641	2.215	4.73E-02
**NQO1**	-0.313	4.664	-2.215	4.73E-02
**GAB1**	-0.300	3.879	-2.215	4.73E-02
**DMKN**	0.298	3.820	2.213	4.75E-02
**OAS2**	-0.529	1.214	-2.212	4.76E-02
**EPHA3**	-0.314	3.050	-2.211	4.77E-02
**TET3**	-0.297	3.312	-2.210	4.78E-02
**DDR2**	-0.295	3.422	-2.210	4.78E-02
**MFSD1**	-0.296	5.791	-2.205	4.82E-02
**ZC3H12C**	-0.316	4.980	-2.202	4.84E-02
**SLC15A1**	-0.296	2.252	-2.200	4.87E-02
**NMUR2**	0.576	-1.755	2.199	4.87E-02
**LAMA4**	-0.297	5.888	-2.198	4.88E-02
**TGFBR1**	-0.413	5.962	-2.198	4.88E-02
**LCN12**	0.595	-0.185	2.197	4.89E-02
**LMO1**	0.305	4.606	2.196	4.90E-02
**GPX8**	-0.291	4.674	-2.194	4.91E-02
**BMPR1B**	-0.523	0.649	-2.194	4.92E-02
**ARHGAP28**	-0.458	0.782	-2.191	4.94E-02
**PI15**	-0.482	2.850	-2.191	4.94E-02
**CYP51A1**	-0.321	6.503	-2.185	4.99E-02
**MRI1**	0.353	2.737	2.185	4.99E-02
**HMCN1**	-0.384	2.221	-2.185	4.99E-02

**MAP3K14**	0.444	2.744	2.185	4.99E-02
**HAUS5**	0.415	1.245	2.185	5.00E-02
**ZMIZ2**	0.298	4.787	2.185	5.00E-02

LogFC: log2 fold change; AveExpr: Average expression across all samples; t: log FC divided its standard error; P.Value: Raw p-value (based on t) from test that log FC differs from 0.

ESM Table 4. DEG list for bulk RNA-seq in ND human islets

	LogFC	AveExpr	t	P.Value
**PAK1**	3.545	6.809	19.535	2.68E-08
**TFF1**	1.348	-0.287	9.028	1.32E-05
**TST**	-0.500	2.686	-5.630	4.13E-04
**S100A9**	-0.586	1.274	-5.254	6.56E-04
**TMSB4X**	-0.419	6.451	-4.673	1.40E-03
**TCN1**	0.506	3.858	4.648	1.45E-03
**PDIA2**	-0.484	3.134	-4.380	2.09E-03
**CELA3B**	-0.609	3.636	-4.338	2.21E-03
**MT1M**	-0.730	0.689	-4.328	2.24E-03
**TSPO**	-0.462	2.626	-4.319	2.27E-03
**TRIP6**	-0.424	3.306	-4.259	2.47E-03
**ANXA2P1**	-0.540	2.180	-4.059	3.28E-03
**TMEM159**	-0.351	2.860	-4.045	3.35E-03
**KRTCAP3**	-0.398	2.682	-4.036	3.40E-03
**VTN**	-0.394	2.996	-4.019	3.48E-03
**TAGLN**	-0.334	4.426	-3.983	3.66E-03
**KY**	0.688	0.656	3.973	3.71E-03
**PTCH1**	0.396	4.080	3.958	3.80E-03
**ORM1**	-0.831	0.574	-3.947	3.86E-03
**CYBA**	-0.404	4.181	-3.936	3.92E-03
**TK1**	-0.556	0.287	-3.908	4.08E-03
**ALDH1A2**	-0.406	1.347	-3.868	4.33E-03
**REG4**	-0.620	-0.046	-3.792	4.84E-03
**SERTAD1**	-0.387	3.058	-3.791	4.85E-03
**LY6E**	-0.358	3.956	-3.758	5.09E-03
**MPST**	-0.329	3.254	-3.735	5.26E-03
**NDUFA4**	-0.328	4.267	-3.704	5.51E-03
**FNDC9**	-0.805	-1.148	-3.645	6.02E-03
**KLK1**	-0.390	2.356	-3.632	6.13E-03
**SLC39A5**	-0.524	1.129	-3.609	6.35E-03
**PLA2G2A**	1.039	1.034	3.608	6.36E-03
**GLRA1**	0.311	4.301	3.594	6.50E-03
**CA4**	-0.426	0.821	-3.591	6.53E-03
**CRIP1**	-0.437	1.349	-3.590	6.53E-03
**SOX10**	-0.869	-0.428	-3.513	7.34E-03
**POLE4**	-0.443	1.104	-3.510	7.36E-03
**CTRC**	-0.486	4.692	-3.455	8.01E-03
**EPO**	-0.898	-1.190	-3.444	8.14E-03
**GPR179**	0.629	-0.852	3.414	8.52E-03
**NEFM**	0.385	6.557	3.407	8.61E-03
**HSD17B2**	-0.488	3.054	-3.398	8.72E-03
**STK17A**	-0.288	4.245	-3.374	9.05E-03
**PROSER2**	-0.381	2.403	-3.363	9.22E-03
**CTXN1**	-0.432	1.558	-3.359	9.26E-03
**RARRES2**	-0.344	3.318	-3.346	9.45E-03
**TSPAN6**	-0.308	3.959	-3.342	9.51E-03
**ACOT9**	-0.293	3.302	-3.327	9.73E-03
**CYP27B1**	-0.409	0.596	-3.302	1.01E-02
**IGFBP1**	-0.747	-0.420	-3.296	1.02E-02

**TSPAN8**	-0.333	5.795	-3.296	1.02E-02
**C1QTNF1**	-0.321	3.201	-3.280	1.05E-02
**IDO1**	2.270	0.221	3.279	1.05E-02
**KAT6B**	0.278	5.826	3.271	1.06E-02
**CELA2A**	-0.654	2.169	-3.267	1.07E-02
**ZNF462**	0.307	4.641	3.253	1.09E-02
**FGD4**	0.305	5.907	3.241	1.11E-02
**SPHKAP**	0.437	1.155	3.226	1.14E-02
**LRRTM2**	0.394	2.662	3.218	1.15E-02
**TNFRSF12A**	-0.305	5.456	-3.211	1.16E-02
**CHMP4A**	-0.323	2.444	-3.208	1.17E-02
**CDC42EP5**	-0.369	2.105	-3.205	1.17E-02
**HTR3A**	-0.447	1.806	-3.175	1.23E-02
**SNHG5**	-0.278	4.716	-3.164	1.25E-02
**ROBO2**	0.270	6.740	3.162	1.26E-02
**CD52**	-0.773	-2.349	-3.159	1.26E-02
**GLDN**	0.569	-0.704	3.133	1.31E-02
**PIK3C2B**	0.322	3.914	3.128	1.32E-02
**HMGN5**	0.312	2.941	3.115	1.35E-02
**CHI3L2**	-0.518	0.492	-3.097	1.39E-02
**LINC00643**	0.254	5.742	3.095	1.39E-02
**DACH1**	0.262	5.156	3.086	1.41E-02
**AADAC**	-0.427	1.370	-3.081	1.42E-02
**ACTG1P20**	-0.341	4.958	-3.061	1.47E-02
**UBA7**	-0.278	3.029	-3.054	1.48E-02
**DEFB1**	-0.308	3.115	-3.028	1.54E-02
**NOV**	-0.454	2.463	-3.027	1.55E-02
**CLDN4**	-0.242	5.867	-3.023	1.56E-02
**KIAA2026**	0.226	5.531	3.022	1.56E-02
**RAB33A**	-0.539	-0.492	-3.021	1.56E-02
**SSC4D**	-0.576	-0.441	-3.020	1.57E-02
**RBFOX3**	0.443	1.604	3.017	1.57E-02
**C1QA**	-0.314	1.724	-3.015	1.58E-02
**CCL3**	-0.472	-0.423	-3.014	1.58E-02
**CCL21**	-1.024	-2.118	-3.011	1.59E-02
**AZGP1**	-0.402	2.725	-2.999	1.62E-02
**CSRNP3**	0.290	5.386	2.999	1.62E-02
**SERPINB3**	0.666	-0.938	2.999	1.62E-02
**RPS3A**	-0.460	3.360	-2.993	1.63E-02
**ZNF827**	0.249	4.172	2.989	1.64E-02
**BHLHB9**	0.295	3.785	2.989	1.64E-02
**WTIP**	-0.476	0.817	-2.978	1.67E-02
**CDKL5**	0.358	2.221	2.973	1.68E-02
**NCOA6**	0.239	5.598	2.972	1.69E-02
**LURAP1L**	-0.261	4.046	-2.966	1.70E-02
**TPD52L1**	-0.273	2.858	-2.963	1.71E-02
**KRT19P2**	-0.367	1.200	-2.957	1.73E-02
**NECTIN3**	0.243	6.339	2.944	1.76E-02
**PYCARD**	-0.426	0.753	-2.943	1.77E-02
**KIAA2022**	0.326	4.508	2.940	1.77E-02
**KRT8**	-0.280	7.305	-2.933	1.79E-02
**PYCRL**	-0.359	1.432	-2.932	1.79E-02

**FGG**	-0.431	5.494	-2.922	1.82E-02
**AMBP**	-0.318	4.626	-2.922	1.83E-02
**RPS7**	-0.316	3.734	-2.920	1.83E-02
**LBP**	-0.738	1.882	-2.913	1.85E-02
**B3GNT7**	-0.290	3.943	-2.906	1.87E-02
**S100A4**	-0.480	0.046	-2.903	1.88E-02
**CLPS**	-0.553	3.707	-2.899	1.89E-02
**ASAP1**	0.330	6.763	2.899	1.89E-02
**MYEOV2**	-0.332	3.915	-2.894	1.91E-02
**CRIP3**	-0.489	0.147	-2.892	1.91E-02
**IGF1**	0.721	-1.690	2.885	1.93E-02
**RASGRF1**	0.248	6.424	2.883	1.94E-02
**AKR1C1**	-0.338	4.418	-2.881	1.95E-02
**HLA-L**	-0.362	0.495	-2.880	1.95E-02
**OCEL1**	-0.265	2.463	-2.876	1.96E-02
**ATXN2**	0.231	5.779	2.870	1.98E-02
**PLXNC1**	0.271	4.950	2.861	2.01E-02
**MTFP1**	-0.309	3.292	-2.860	2.01E-02
**CAPG**	-0.292	3.012	-2.858	2.02E-02
**IFIT1**	0.266	3.633	2.850	2.05E-02
**LYPD1**	-0.323	4.198	-2.847	2.05E-02
**SLC38A5**	-0.531	2.358	-2.847	2.05E-02
**MYH10**	0.261	7.430	2.845	2.06E-02
**PLCXD3**	0.256	8.210	2.837	2.09E-02
**LOC100130331**	-0.366	3.161	-2.836	2.09E-02
**SMO**	-0.314	2.197	-2.834	2.10E-02
**MNX1**	0.227	4.360	2.833	2.10E-02
**ALDH1L1**	-0.422	1.173	-2.829	2.11E-02
**JOSD2**	-0.319	2.265	-2.829	2.11E-02
**MEIS2**	0.208	7.215	2.829	2.11E-02
**RAB32**	-0.304	3.683	-2.827	2.12E-02
**RPS21**	-0.321	5.707	-2.820	2.14E-02
**UGT2B7**	-0.388	1.605	-2.814	2.16E-02
**FMO1**	-0.494	-0.144	-2.813	2.17E-02
**FRMPD1**	0.314	1.805	2.811	2.17E-02
**FUNDC2**	-0.229	4.939	-2.804	2.20E-02
**RUNX1T1**	0.318	4.118	2.803	2.20E-02
**FAM109A**	-0.262	3.167	-2.797	2.22E-02
**TSHZ1**	0.247	5.902	2.797	2.22E-02
**KIF13A**	0.254	6.212	2.796	2.22E-02
**GPR143**	-0.510	0.157	-2.793	2.24E-02
**SERPINA5**	-0.259	5.963	-2.792	2.24E-02
**DUOXA1**	-0.636	-1.127	-2.792	2.24E-02
**RPL29**	-0.307	5.680	-2.790	2.25E-02
**TGFBI**	-0.302	7.350	-2.786	2.26E-02
**CCL18**	-0.487	-0.551	-2.783	2.27E-02
**MINOS1P1**	-0.373	2.021	-2.775	2.30E-02
**FNDC4**	-0.306	3.447	-2.774	2.30E-02
**ZNRD1**	-0.276	2.639	-2.774	2.30E-02
**FRRS1L**	0.374	1.719	2.772	2.31E-02
**BCOR**	0.220	5.501	2.766	2.33E-02
**MFSD7**	-0.392	0.850	-2.766	2.33E-02

**BMP5**	0.241	6.096	2.762	2.35E-02
**SPHK1**	-0.280	3.581	-2.756	2.37E-02
**KCNK15**	-0.348	1.019	-2.755	2.37E-02
**DUSP5**	-0.287	4.789	-2.748	2.40E-02
**TPRN**	-0.255	2.782	-2.748	2.40E-02
**F2RL2**	0.395	1.735	2.747	2.40E-02
**VTCN1**	-0.275	3.887	-2.744	2.42E-02
**ROMO1**	-0.320	4.357	-2.742	2.43E-02
**SLC17A6**	0.204	4.980	2.740	2.43E-02
**PLEKHJ1**	-0.331	3.575	-2.725	2.49E-02
**MMP9**	-0.412	3.754	-2.725	2.49E-02
**CARTPT**	-0.941	-1.145	-2.722	2.50E-02
**CLEC4E**	-0.569	0.653	-2.722	2.50E-02
**MYDGF**	-0.237	5.623	-2.720	2.51E-02
**CD151**	-0.222	6.622	-2.716	2.53E-02
**APC**	0.315	6.467	2.711	2.54E-02
**MT1G**	-0.267	3.219	-2.707	2.56E-02
**CXCL10**	2.517	1.029	2.707	2.56E-02
**REEP6**	-0.343	2.186	-2.703	2.58E-02
**ACSM1**	-0.353	0.829	-2.701	2.59E-02
**IFI27**	0.578	0.340	2.698	2.60E-02
**HOMER3**	-0.273	2.432	-2.697	2.60E-02
**FGL1**	-0.364	3.539	-2.694	2.61E-02
**EFNA4**	-0.446	0.773	-2.694	2.62E-02
**RPL9**	-0.259	3.564	-2.685	2.66E-02
**S100A13**	-0.303	2.794	-2.677	2.69E-02
**ROBO1**	0.269	5.958	2.676	2.69E-02
**KIRREL3**	0.279	3.871	2.675	2.70E-02
**COL8A2**	-0.446	-0.235	-2.672	2.71E-02
**FOXA3**	-0.273	3.904	-2.669	2.72E-02
**TNRC6B**	0.265	5.921	2.668	2.72E-02
**CNKSR2**	0.293	3.606	2.668	2.72E-02
**TRADD**	-0.316	2.475	-2.665	2.74E-02
**KRT18**	-0.289	6.407	-2.660	2.76E-02
**ENPP1**	0.209	5.200	2.658	2.77E-02
**NFASC**	0.250	7.079	2.654	2.79E-02
**TEAD2**	-0.248	4.045	-2.653	2.79E-02
**SPINK1**	-0.358	8.296	-2.651	2.80E-02
**AKR7A3**	-0.462	1.555	-2.646	2.82E-02
**SMIM22**	-0.361	3.199	-2.640	2.85E-02
**KRT80**	-0.287	3.854	-2.639	2.86E-02
**SNX27**	0.229	5.374	2.627	2.91E-02
**SRGAP2D**	0.570	0.377	2.626	2.91E-02
**CRISP3**	0.647	-1.150	2.622	2.93E-02
**TM4SF5**	-0.671	-2.346	-2.619	2.94E-02
**ERP27**	-0.269	3.027	-2.613	2.97E-02
**LCORL**	0.223	4.755	2.612	2.98E-02
**TMEM98**	-0.266	3.591	-2.608	3.00E-02
**LINC01128**	0.223	4.537	2.606	3.01E-02
**NR0B2**	-0.330	2.076	-2.606	3.01E-02
**PRSS3P2**	-0.410	7.297	-2.604	3.02E-02
**PGAM1**	-0.286	3.592	-2.603	3.02E-02

**CDC42EP2**	-0.255	3.092	-2.602	3.03E-02
**FUT2**	0.266	2.025	2.602	3.03E-02
**SLC25A34**	0.246	4.113	2.602	3.03E-02
**NFKBIE**	-0.248	3.104	-2.602	3.03E-02
**CYB561D2**	-0.319	3.016	-2.600	3.04E-02
**TMEM255A**	0.338	2.068	2.600	3.04E-02
**CPB1**	-0.314	7.451	-2.599	3.04E-02
**GPC4**	-0.292	3.398	-2.597	3.05E-02
**SLC4A8**	0.272	6.270	2.596	3.05E-02
**PNLIPRP1**	-0.354	4.721	-2.593	3.07E-02
**CXCL11**	2.278	-0.165	2.592	3.08E-02
**SIGIRR**	-0.234	2.928	-2.591	3.08E-02
**F2RL1**	-0.240	4.703	-2.587	3.10E-02
**PSMB10**	-0.296	3.923	-2.577	3.15E-02
**NCOA2**	0.220	5.097	2.574	3.16E-02
**JARID2**	0.241	4.230	2.574	3.17E-02
**AKR1C3**	-0.284	5.330	-2.573	3.17E-02
**MAGIX**	-0.321	1.390	-2.572	3.18E-02
**MGST1**	-0.228	4.707	-2.566	3.20E-02
**SLC43A1**	-0.311	2.805	-2.560	3.23E-02
**CASR**	0.240	7.145	2.560	3.24E-02
**LBX2-AS1**	-0.503	-0.037	-2.559	3.24E-02
**PDZK1IP1**	-0.275	4.637	-2.556	3.26E-02
**GEMIN7**	-0.246	2.785	-2.556	3.26E-02
**NEFH**	0.319	2.224	2.552	3.28E-02
**RPL23P8**	-0.367	2.822	-2.549	3.29E-02
**DAPP1**	-0.482	-0.217	-2.544	3.32E-02
**ADAMTSL1**	0.353	1.980	2.542	3.33E-02
**VIM**	-0.231	8.528	-2.542	3.33E-02
**NDUFA11**	-0.433	2.614	-2.538	3.35E-02
**PCDHB14**	0.238	3.323	2.538	3.35E-02
**SOX5**	0.305	3.071	2.533	3.38E-02
**NEUROD1**	0.212	7.124	2.533	3.38E-02
**CXCL5**	-0.506	4.236	-2.532	3.38E-02
**MTMR14**	-0.260	3.503	-2.531	3.39E-02
**XKR7**	0.379	1.689	2.529	3.40E-02
**CCDC170**	0.461	-0.889	2.526	3.42E-02
**ACTG1**	-0.265	10.457	-2.524	3.43E-02
**CLDN7**	-0.235	6.413	-2.522	3.44E-02
**NALCN**	0.232	4.192	2.522	3.44E-02
**ITIH4**	-0.253	3.542	-2.519	3.45E-02
**ZNF33A**	0.234	5.863	2.517	3.47E-02
**MUC5B**	1.354	-1.442	2.515	3.48E-02
**RPS19**	-0.286	6.643	-2.514	3.48E-02
**PGR**	0.255	5.241	2.514	3.48E-02
**MAP1B**	0.251	8.174	2.504	3.54E-02
**PLEKHA6**	0.266	6.590	2.503	3.54E-02
**ATRX**	0.290	6.816	2.503	3.54E-02
**RPS12**	-0.330	5.573	-2.495	3.59E-02
**PER3**	0.250	5.385	2.490	3.62E-02
**WNK1**	0.203	7.209	2.489	3.62E-02
**HTR1F**	0.356	2.231	2.486	3.64E-02

**SELM**	-0.251	4.205	-2.482	3.66E-02
**HSPB7**	-0.576	0.345	-2.482	3.66E-02
**RCAN3**	0.194	5.837	2.481	3.67E-02
**C4BPB**	-0.399	0.148	-2.481	3.67E-02
**CCL11**	0.621	-0.674	2.481	3.67E-02
**PHYHD1**	-0.437	-0.040	-2.480	3.67E-02
**CDR2L**	-0.234	3.569	-2.479	3.68E-02
**LOC285484**	0.311	1.885	2.478	3.69E-02
**PTPRJ**	0.204	6.178	2.477	3.69E-02
**NAV1**	0.231	7.034	2.477	3.69E-02
**CBS**	-0.220	3.351	-2.476	3.70E-02
**MAP1A**	0.225	6.098	2.475	3.71E-02
**CITED4**	-0.299	2.443	-2.474	3.71E-02
**LGALS2**	-0.320	3.136	-2.473	3.72E-02
**KIF21A**	0.258	6.423	2.472	3.72E-02
**ZSWIM1**	0.427	2.342	2.472	3.72E-02
**COMMD5**	-0.273	2.983	-2.471	3.73E-02
**ATP2B2**	0.407	-0.590	2.469	3.74E-02
**FAM21C**	0.324	2.815	2.468	3.75E-02
**PPCDC**	-0.281	1.735	-2.468	3.75E-02
**MAN1C1**	0.197	5.855	2.467	3.75E-02
**RSG1**	-0.350	0.898	-2.466	3.76E-02
**ZC3H13**	0.253	5.656	2.466	3.76E-02
**FGFBP1**	-0.438	0.066	-2.466	3.76E-02
**GGT1**	-0.226	3.774	-2.466	3.76E-02
**GNL3L**	0.215	4.705	2.466	3.76E-02
**CTRB1**	-0.424	4.915	-2.463	3.78E-02
**UGT1A6**	-0.433	0.876	-2.461	3.79E-02
**LRMP**	-0.503	-0.407	-2.461	3.79E-02
**TNRC6C**	0.342	3.478	2.461	3.79E-02
**PLCH1**	0.229	3.383	2.459	3.80E-02
**MOB1B**	0.317	7.295	2.459	3.80E-02
**S100A16**	-0.273	3.886	-2.459	3.80E-02
**AKR1B10**	-0.378	4.568	-2.456	3.82E-02
**SFMBT2**	0.270	3.642	2.453	3.84E-02
**COMMD4**	-0.247	3.250	-2.446	3.88E-02
**GPR148**	0.453	-0.061	2.446	3.88E-02
**ZNF626**	0.206	3.581	2.446	3.88E-02
**TRNAU1AP**	-0.236	2.781	-2.445	3.89E-02
**LGALS1**	-0.289	5.481	-2.444	3.89E-02
**PCDH7**	0.229	5.475	2.444	3.89E-02
**OSBP2**	0.273	2.578	2.442	3.91E-02
**PLEKHG6**	-0.263	2.629	-2.437	3.93E-02
**OPA3**	0.251	4.456	2.436	3.94E-02
**SQRDL**	-0.241	4.716	-2.436	3.94E-02
**ZKSCAN1**	0.224	6.794	2.434	3.95E-02
**MZT2B**	-0.284	4.286	-2.431	3.97E-02
**KCNJ2**	0.246	3.054	2.430	3.98E-02
**IL4I1**	-0.337	0.708	-2.429	3.99E-02
**EPN3**	-0.312	1.762	-2.429	3.99E-02
**SLC31A2**	-0.265	2.598	-2.426	4.00E-02
**SOBP**	0.183	4.998	2.423	4.02E-02

**AQP1**	-0.323	5.236	-2.418	4.06E-02
**OPN3**	-0.282	3.605	-2.417	4.06E-02
**FGA**	-0.386	4.873	-2.416	4.07E-02
**TPTEP1**	-0.639	-1.879	-2.416	4.07E-02
**GOLIM4**	0.243	4.639	2.415	4.07E-02
**CARHSP1**	-0.210	5.135	-2.411	4.10E-02
**BANF1**	-0.289	4.001	-2.410	4.11E-02
**AKAP9**	0.402	6.519	2.410	4.11E-02
**HHIP**	-0.527	-2.318	-2.407	4.13E-02
**ZNF219**	-0.203	3.680	-2.405	4.14E-02
**ARHGAP8**	-1.111	-2.641	-2.399	4.18E-02
**SLC24A2**	0.441	1.207	2.398	4.19E-02
**RTN4RL1**	0.243	3.701	2.398	4.19E-02
**BAHCC1**	0.259	3.295	2.397	4.20E-02
**RPL10**	-0.246	6.801	-2.396	4.20E-02
**FAM96B**	-0.234	4.107	-2.396	4.20E-02
**ASL**	-0.248	4.053	-2.396	4.20E-02
**NFIC**	0.201	7.346	2.394	4.22E-02
**ZNF568**	0.234	3.800	2.391	4.23E-02
**ANKS1B**	0.270	2.707	2.391	4.24E-02
**CPT1A**	0.323	5.280	2.390	4.24E-02
**MCC**	0.224	5.279	2.390	4.24E-02
**TRPV6**	-0.210	3.749	-2.389	4.25E-02
**VAMP5**	-0.355	1.039	-2.388	4.26E-02
**GJB3**	-0.499	-0.288	-2.387	4.26E-02
**PPP4C**	-0.222	5.043	-2.387	4.26E-02
**LOC100335030**	-0.736	-0.821	-2.387	4.26E-02
**ZFHX3**	0.273	5.473	2.386	4.27E-02
**KCNA3**	0.232	2.902	2.385	4.27E-02
**SSTR2**	0.230	5.229	2.380	4.31E-02
**HIPK2**	0.222	7.342	2.380	4.31E-02
**POLR2H**	-0.209	3.867	-2.380	4.31E-02
**RAB3B**	0.208	7.786	2.378	4.33E-02
**PDE1B**	-0.310	2.163	-2.376	4.34E-02
**EPB41L3**	0.191	6.749	2.376	4.34E-02
**VDAC2**	-0.287	3.736	-2.376	4.34E-02
**KCNG3**	0.243	3.368	2.375	4.35E-02
**UPP1**	-0.356	2.552	-2.373	4.36E-02
**PDS5B**	0.204	5.575	2.373	4.36E-02
**ESRRA**	-0.231	4.200	-2.370	4.38E-02
**RNF180**	0.217	4.137	2.368	4.40E-02
**TCEAL2**	0.202	4.407	2.365	4.42E-02
**TOR4A**	-0.278	2.342	-2.364	4.42E-02
**PPIG**	0.230	5.563	2.364	4.42E-02
**CCDC158**	0.342	1.188	2.363	4.43E-02
**KIF5C**	0.261	7.784	2.359	4.45E-02
**CPTP**	-0.280	2.882	-2.359	4.46E-02
**ZFAS1**	-0.219	4.899	-2.358	4.46E-02
**CLEC11A**	-0.280	1.823	-2.357	4.47E-02
**FAM195A**	-0.267	2.541	-2.357	4.47E-02
**B3GALT2**	0.290	3.571	2.356	4.48E-02
**ZNF280B**	0.316	2.344	2.356	4.48E-02

**CEL**	-0.385	5.954	-2.355	4.48E-02
**ABTB1**	-0.251	2.072	-2.355	4.49E-02
**SLC44A4**	-0.184	5.272	-2.351	4.52E-02
**HLA-DRB6**	-0.379	-0.055	-2.350	4.52E-02
**LMO3**	0.248	2.136	2.347	4.55E-02
**BLVRB**	-0.246	3.804	-2.345	4.56E-02
**RPLP2**	-0.325	6.400	-2.344	4.56E-02
**AKR1B1**	-0.245	5.161	-2.341	4.59E-02
**SNRPB**	-0.221	5.494	-2.340	4.59E-02
**MGC27345**	0.301	1.966	2.339	4.60E-02
**RERE**	0.178	6.581	2.339	4.60E-02
**TSPAN15**	-0.214	4.495	-2.339	4.60E-02
**TMEM120A**	-0.242	3.547	-2.335	4.63E-02
**IL33**	-0.801	1.866	-2.333	4.64E-02
**LONRF2**	0.280	5.928	2.333	4.65E-02
**FUT6**	-0.389	0.336	-2.331	4.66E-02
**ITGAE**	-0.266	2.148	-2.330	4.67E-02
**BCAS1**	0.673	-1.575	2.329	4.67E-02
**GPRC5B**	-0.211	4.659	-2.329	4.68E-02
**DMKN**	-0.193	3.797	-2.326	4.70E-02
**TMEM134**	-0.245	2.796	-2.326	4.70E-02
**CXXC4**	0.221	5.084	2.326	4.70E-02
**TCF4**	0.189	5.546	2.324	4.71E-02
**IL23A**	-0.412	0.112	-2.322	4.72E-02
**TMEM102**	-0.287	2.168	-2.322	4.73E-02
**LMNA**	-0.317	6.500	-2.322	4.73E-02
**CTRL**	-0.445	0.312	-2.321	4.73E-02
**RHOD**	-0.287	2.569	-2.317	4.77E-02
**ERICH3**	0.275	2.224	2.317	4.77E-02
**PLEKHM3**	0.186	4.669	2.315	4.78E-02
**EIF2AK2**	0.200	4.809	2.313	4.80E-02
**TSHZ3**	0.202	4.253	2.311	4.81E-02
**ZNF326**	0.258	3.945	2.311	4.81E-02
**HIVEP3**	0.212	3.756	2.310	4.82E-02
**KRT7**	-0.315	6.382	-2.309	4.83E-02
**DOCK10**	0.240	4.931	2.308	4.84E-02
**KIAA1211**	0.331	3.573	2.307	4.84E-02
**SLTM**	0.252	6.074	2.306	4.85E-02
**ANO3**	-0.493	-0.306	-2.306	4.85E-02
**HSPB8**	-0.223	3.982	-2.304	4.87E-02
**PLCL2**	0.171	5.491	2.303	4.87E-02
**C4orf48**	-0.392	2.167	-2.302	4.88E-02
**ALDH16A1**	-0.250	3.398	-2.301	4.89E-02
**ANXA9**	-0.296	1.671	-2.301	4.89E-02
**KSR2**	0.290	3.810	2.300	4.90E-02
**TOX**	0.178	4.712	2.300	4.90E-02
**PALM2**	0.229	3.572	2.298	4.91E-02
**NEGR1**	0.203	4.874	2.296	4.93E-02
**PCLO**	0.367	5.680	2.296	4.93E-02
**RELB**	-0.217	3.837	-2.295	4.94E-02
**CACNA1C**	0.290	4.994	2.295	4.94E-02
**ZC3HAV1**	0.194	6.106	2.295	4.94E-02

**JAKMIP2**	0.276	4.017	2.294	4.95E-02
**NCOR1**	0.202	6.488	2.292	4.96E-02
**TRIM47**	-0.254	3.625	-2.291	4.97E-02
**TYRO3**	-0.374	0.980	-2.291	4.97E-02
**DOC2B**	0.893	-1.156	2.290	4.98E-02
**NFATC2**	0.258	3.282	2.289	4.98E-02
**RGL1**	0.179	5.394	2.288	4.99E-02

LogFC: log2 fold change; AveExpr: Average expression across all samples; t: log FC divided its standard error; P.Value: Raw p-value (based on t) from test that log FC differs from 0.

ESM Table 5. Fasting plasma insulin and blood glucose levels in HFD-control and -*i*βPAK1-Tg mice vs. chow-fed littermate mice.

	Ctrl: sTg Chow + Dox n=9	Ctrl: dTg HFD n=8	*ι*βPAK1-Tg HFD + Dox n=10
Plasma insulin (ng/ml)	0.57 ± 0.08	0.90 ± 0.07 *	0.54 ± 0.07 [a]
Blood glucose (mmol/l)	10.64 ± 0.41	12.20 ± 0.59	10.90 ± 0.51

Plasma was collected and blood glucose was measured from 6-h fasted 15-week-old male mice. Data represent the mean ± SEM; [a],*$p<0.05$; one-way ANOVA, Tukey's comparisons test. (*) Ctrl; sTg-Chow + Dox vs. Ctrl; dTg-HFD. ([a]) Ctrl; sTg-Chow + Dox vs. *i*βPAK1-Tg-HFD+ Dox.

ESM Table 6. Tissue weight analysis (% of body weight)

	Ctrl: sTg<br>Chow + Dox<br>n=7	Ctrl: dTg<br>HFD<br>n=8	iβPAK1-Tg<br>HFD + Dox<br>n=11
Body Weight (g)	26.7 ± 1	32.3 ± 1*	33.6 ± 1***
		Tissue (% of body weight)	
Liver	4.10 ± 0.15	3.54 ± 0.14*	3.74 ± 0.13
Heart	0.57 ± 0.08	0.56 ± 0.01	0.52 ± 0.05
Fat	1.71 ± 0.18	3.84 ± 0.39**	4.25 ± 0.40***
Skeletal Muscle	2.17 ± 0.16	1.73 ± 0.19	1.54 ± 0.16*
Kidney	1.37 ± 0.08	1.18 ± 0.07	1.08 ± 0.05
Spleen	0.19 ± 0.05	0.25 ± 0.03	0.23 ± 0.03
Lung	0.61 ± 0.06	0.53 ± 0.03	0.47 ± 0.06

Tissues were collected from 18-week-old male transgenic mice. Data represent the mean ± SEM. No significant differences were detected between HFD-fed Ctrl: dTg and iβPAK1-Tg. *p<0.05, **p<0.01, and ***p<0.001; one-way ANOVA, Tukey's comparisons test between Ctrl; sTg-Chow + Dox and Ctrl; dTg-HFD, and iβPAK1-Tg-HFD+ Dox.

<u>ESM Table 7.</u> Fasting plasma analytes HFD-control and iβPAK1-Tg mice vs. chow-fed littermate mice

	Ctrl: sTg<br>Chow + Dox<br>n=9	Ctrl: dTg<br>HFD<br>n=8	iβPAK1-Tg<br>HFD + Dox<br>n=10
Cholesterol (mg/ml)	0.97 ± 0.04	1.91 ± 0.08 ***	2.02 ± 0.05 ***
Triglycerides (mg/ml)	0.25 ± 0.06	0.24 ± 0.02	0.21 ± 0.03
NEFA (mmol/l)	0.69 ± 0.08	0.76 ± 0.10	0.82 ± 0.07

Plasma was collected from 16-h fasted 18-week-old male transgenic mice. NEFA, Non-esterified fatty acid. Data represent the mean ± SEM. No significant differences were detected between HFD-fed Ctrl: dTg and iβPAK1-Tg. ***p<0.001; one-way ANOVA, Tukey's comparisons test between Ctrl; sTg-Chow + Dox and Ctrl; dTg-HFD, and iβPAK1-Tg-HFD+ Dox.

ESM Figure 1. Transcriptomic analysis of PAK1-enriched human islets

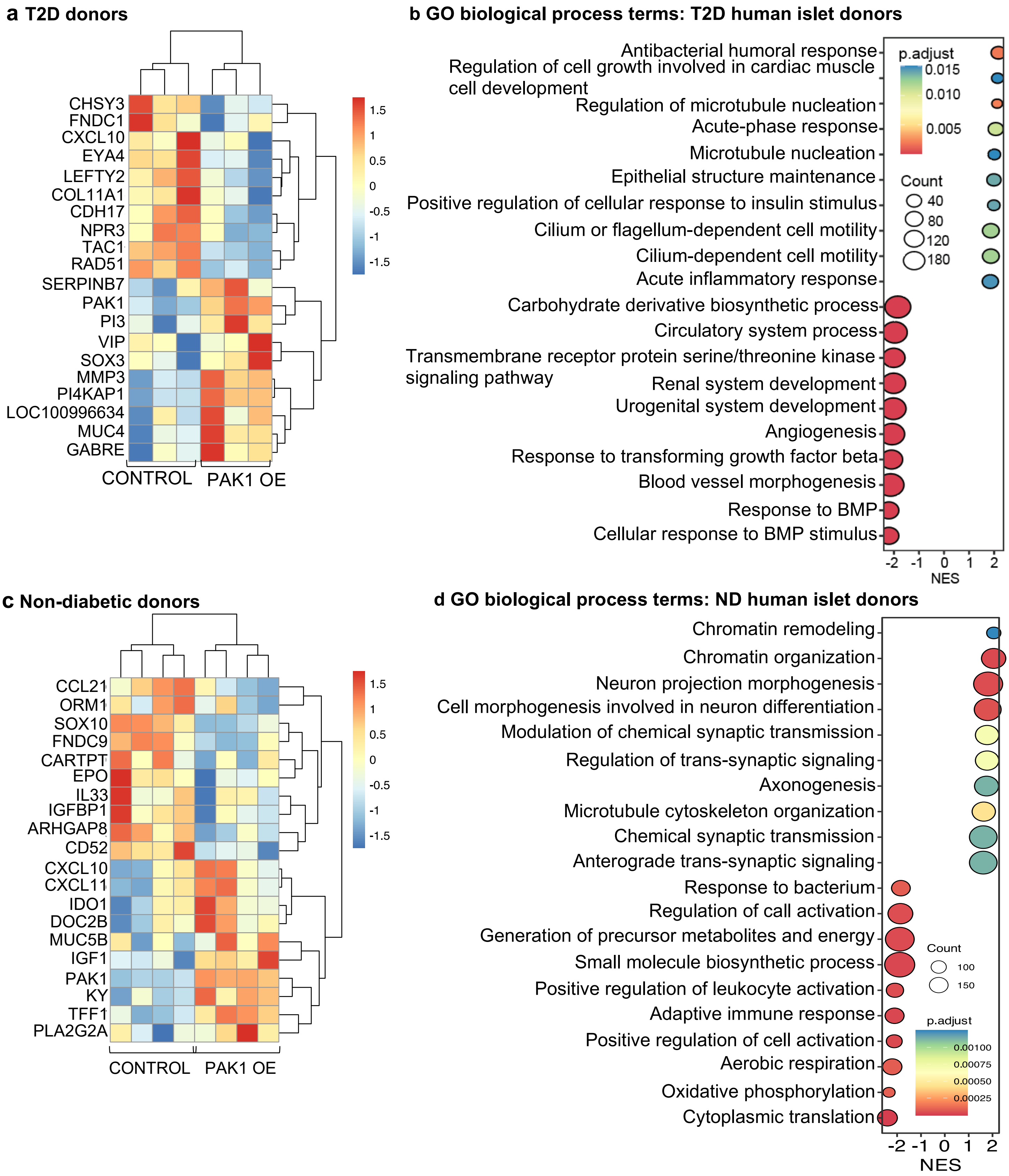

ESM Fig. 1. Heat map represents top genes regulated by PAK1 in T2D (a) and ND (c) human islets. Up- or down-regulated top 10 pathways in PAK1-transduced T2D (b) and ND (d) human islets. The size of circles is proportional to the number of genes included in each pathway. The colour indicates statistical confidence.

ESM Figure 2. Validation of purity of the nuclear and non-nuclear fractions

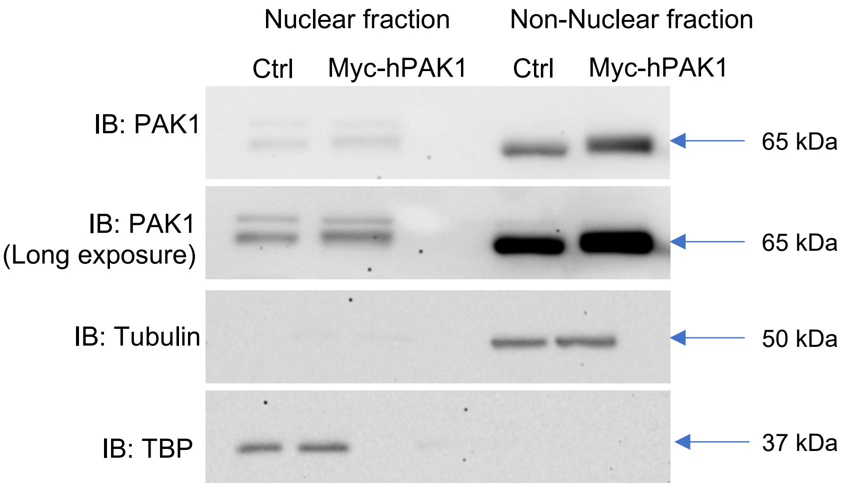

ESM Fig. 2. (a) Purity of the nuclear and non-nuclear fractions. Nuclear and non-nuclear fractions were evaluated for purity using TBP as the nuclear marker, and tubulin for the non-nuclear fraction.

ESM Figure 3. IPA and HOMER analyses in human EndoC-βH1 cells

a

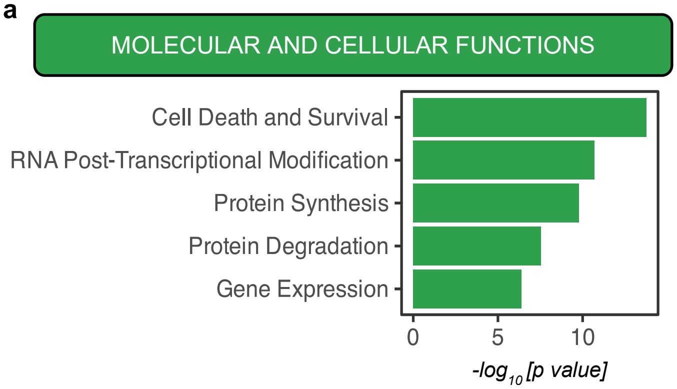

b

Rank	Motif	P value	Representative TF
1	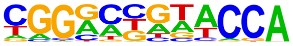	1×10^{-86}	PRDM10
2	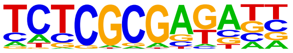	1×10^{-57}	ZBTB33
3	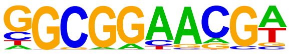	1×10^{-26}	HINFP
4	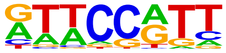	1×10^{-17}	NFATC1
5	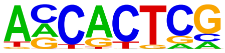	1×10^{-17}	BAPX1

ESM Fig. 3. (a) Top molecular and cellular function analysis using ingenuity Pathway analysis (IPA). (b) Top enriched DNA motifs from HOMER. The best Transcription factor (TF) match representing each motif is listed.

ESM Figure 4. PAK1 overexpression does not alter transcription levels of insulin biogenesis related genes under basal conditions (5.5 mmol/l glucose)

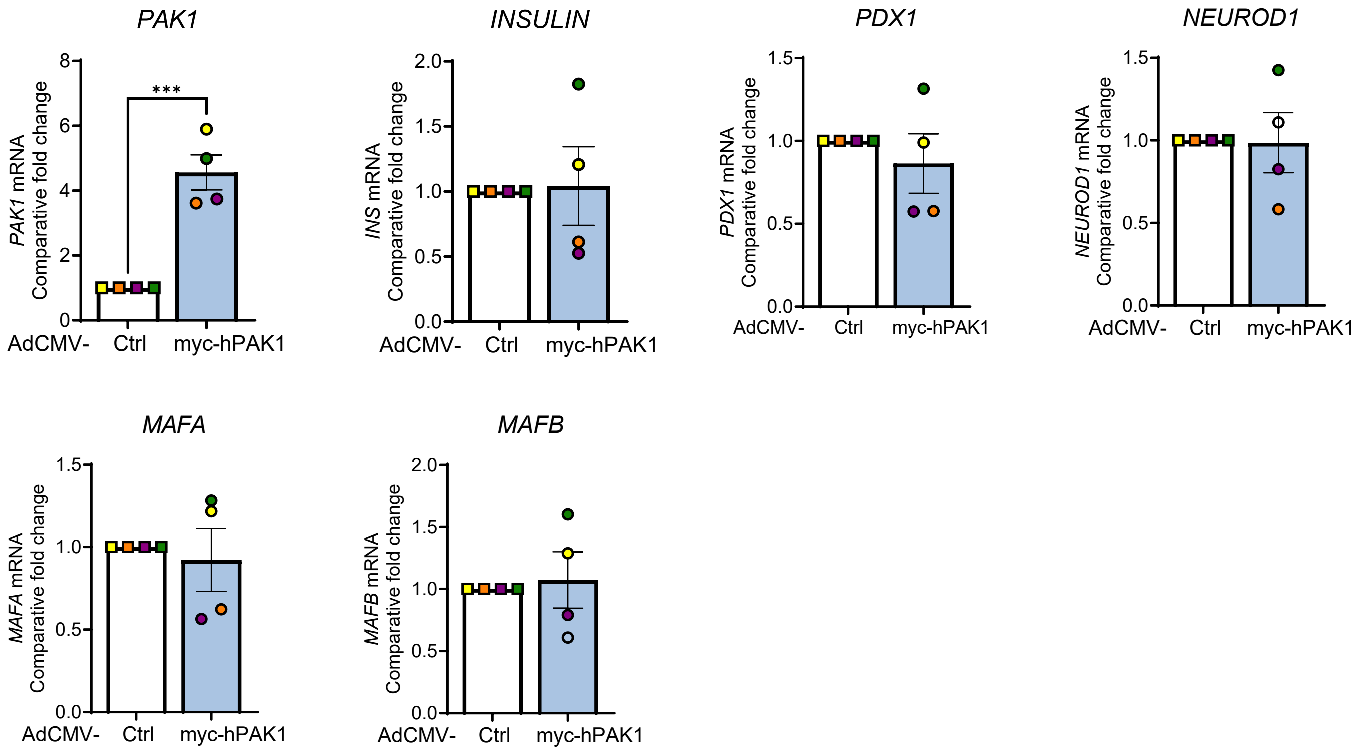

ESM Fig. 4. Human EndoC-βH1 cells were transduced with 100 moi of Ad-CMV myc-h*PAK1* or control for 48 h. Isolated RNA was used for qPCR analysis of *INS, Pdx1, NEUROD1, MAFA*, and *MAFB* levels. All data are shown mean ±SEM. ***p<0.001 (unpaired two-tailed Student's t-test).

ESM Figure 5. Beta cell-specific PAK1 enrichment enhances glucose tolerance

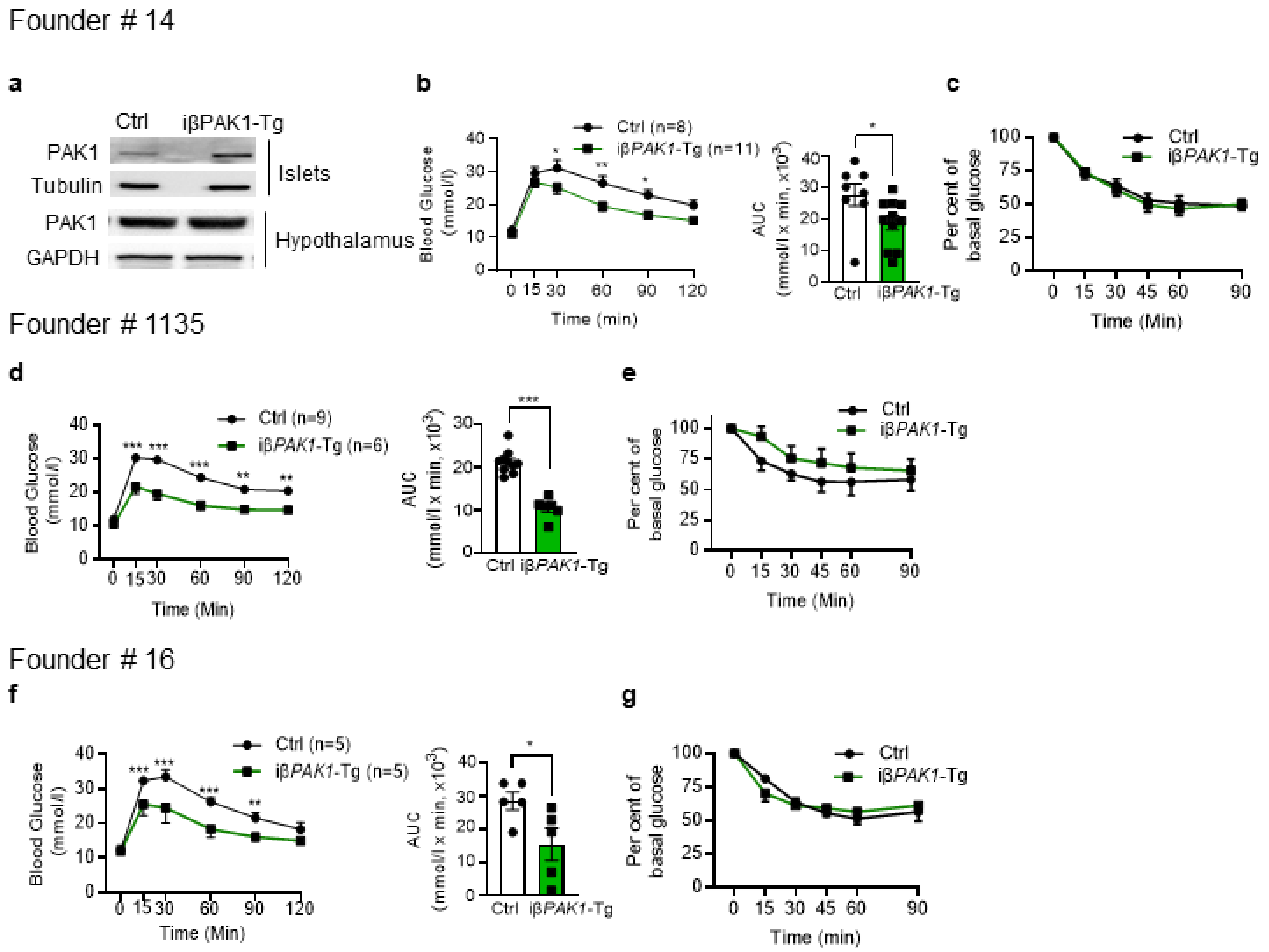

ESM Fig. 5. Nine-week-old mice were administered chow diet with doxycycline (Purina Rodent Chow with 600 mg/Kg of Dox) for 3 weeks prior to experiments to yield stable transgene expression. (a) Beta cell-specific PAK1 enrichment was demonstrated by immunoblot. (b) Significantly improved glucose tolerance in iβPAK1-Tg mice with Dox-induction compared with control mice without Dox-induction (founder line #14, City of Hope). Ctrl mice n=8, iβPAK1-Tg mice n=11. AUC of IPGTT curve. (c) IPITT. (d-g) IPGTT (d, f) and IPITT (e, g) testing of Ctrl (-Dox) or iβPAK1-Tg (+ Dox) of the other two founder lines (#1135 from IUSM and #16 from City of Hope) (n=5-8). Data are shown as the mean ± SEM. *$p<0.05$, **$p<0.01$, ***$p<0.001$ (unpaired two-tailed Student's t-test).

<u>ESM Figure 6.</u> Beta cell-specific PAK1 enrichment in high-fat diet (HFD)-fed mice

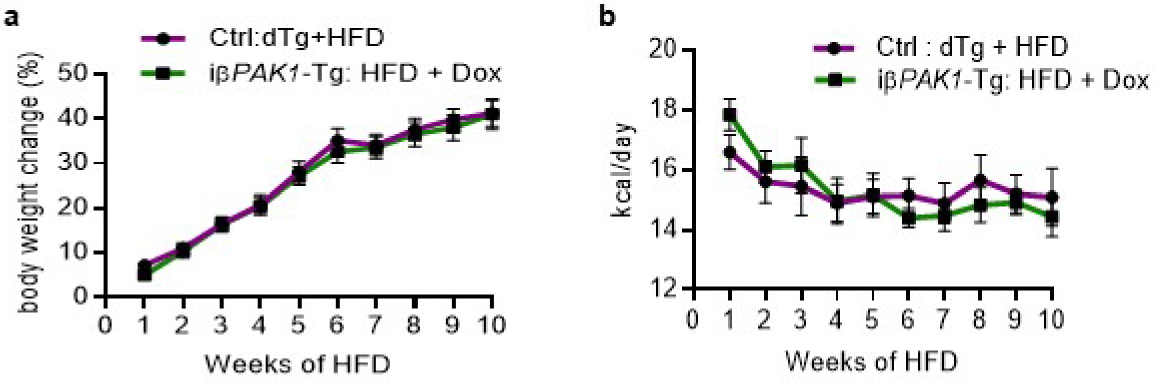

ESM Fig. 6. (a) Cumulative percent increase in body weight in Ctrl (dTg + HFD, n=8) and iβPAK1-Tg (HFD + Dox, n=11) mice. (b) Average daily calorie consumption per week over the HFD feeding period. Data are shown as the mean ± SEM. No significant differences were detected between HFD-fed Ctrl: dTg and iβPAK1-Tg. Statistical significance was evaluated using unpaired two-tailed Student’s t-test.

ESM Figure 7. Diabetes-related gene and pathway analysis of PAK1-enriched ND and T2D human islets

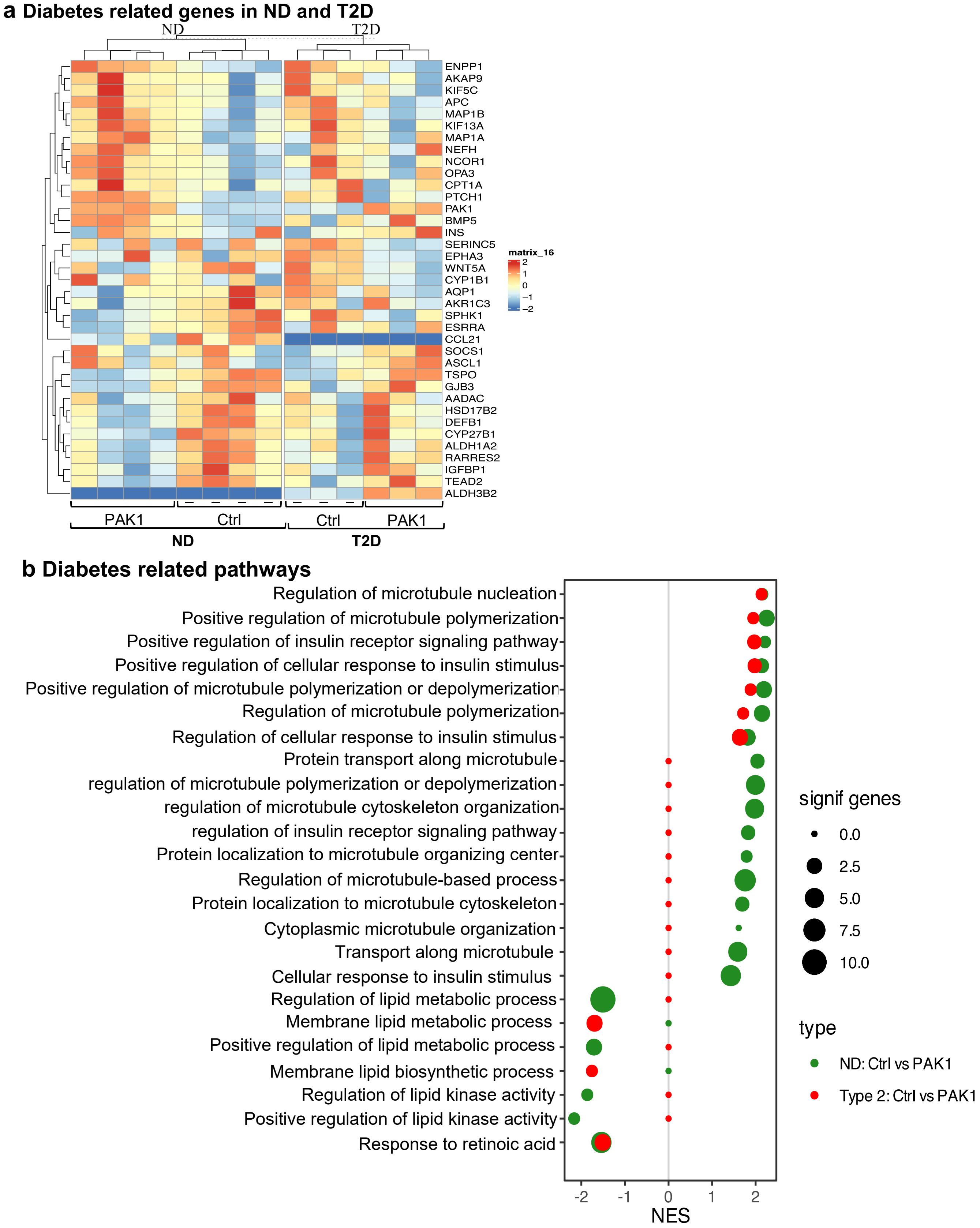

ESM Fig. 7. (a) Heat map represents diabetes-related genes in ND and T2D human islets transduced with control (Ctrl) or PAK1 adenoviruses. (b) Diabetes-related pathways are displayed side-by-side in the ND and Type 2 human islets RNA-seq analysis. The size of the circles corresponds to the number of DEGs.
